# The Continuum of Aging and Age-Related Diseases: Common Mechanisms but Different Rates

**DOI:** 10.3389/fmed.2018.00061

**Published:** 2018-03-12

**Authors:** Claudio Franceschi, Paolo Garagnani, Cristina Morsiani, Maria Conte, Aurelia Santoro, Andrea Grignolio, Daniela Monti, Miriam Capri, Stefano Salvioli

**Affiliations:** ^1^Institute of Neurological Sciences, University of Bologna, Bellaria Hospital, Bologna, Italy; ^2^Department of Experimental, Diagnostic and Specialty Medicine (DIMES), University of Bologna, Bologna, Italy; ^3^Clinical Chemistry, Department of Laboratory Medicine, Karolinska Institutet at Huddinge University Hospital, Stockholm, Sweden; ^4^Applied Biomedical Research Center (CRBA), S. Orsola-Malpighi Polyclinic, Bologna, Italy; ^5^CNR Institute of Molecular Genetics, Unit of Bologna, Bologna, Italy; ^6^Interdepartmental Center “L. Galvani” (CIG), University of Bologna, Bologna, Italy; ^7^Unit and Museum of History of Medicine, Department of Molecular Medicine, Sapienza University of Rome, Rome, Italy; ^8^Department of Experimental and Clinical Biomedical Sciences “Mario Serio”, University of Florence, Florence, Italy

**Keywords:** aging, longevity, age-related diseases, inflammaging, biomarkers, geroscience

## Abstract

Geroscience, the new interdisciplinary field that aims to understand the relationship between aging and chronic age-related diseases (ARDs) and geriatric syndromes (GSs), is based on epidemiological evidence and experimental data that aging is the major risk factor for such pathologies and assumes that aging and ARDs/GSs share a common set of basic biological mechanisms. A consequence is that the primary target of medicine is to combat aging instead of any single ARD/GSs one by one, as favored by the fragmentation into hundreds of specialties and sub-specialties. If the same molecular and cellular mechanisms underpin both aging and ARDs/GSs, a major question emerges: which is the difference, if any, between aging and ARDs/GSs? The hypothesis that ARDs and GSs such as frailty can be conceptualized as accelerated aging will be discussed by analyzing in particular frailty, sarcopenia, chronic obstructive pulmonary disease, cancer, neurodegenerative diseases such as Alzheimer and Parkinson as well as Down syndrome as an example of progeroid syndrome. According to this integrated view, aging and ARDs/GSs become part of a continuum where precise boundaries do not exist and the two extremes are represented by centenarians, who largely avoided or postponed most ARDs/GSs and are characterized by decelerated aging, and patients who suffered one or more severe ARDs in their 60s, 70s, and 80s and show signs of accelerated aging, respectively. In between these two extremes, there is a continuum of intermediate trajectories representing a sort of gray area. Thus, clinically different, classical ARDs/GSs are, indeed, the result of peculiar combinations of alterations regarding the same, limited set of basic mechanisms shared with the aging process. Whether an individual will follow a trajectory of accelerated or decelerated aging will depend on his/her genetic background interacting lifelong with environmental and lifestyle factors. If ARDs and GSs are manifestations of accelerated aging, it is urgent to identify markers capable of distinguishing between biological and chronological age to identify subjects at higher risk of developing ARDs and GSs. To this aim, we propose the use of DNA methylation, N-glycans profiling, and gut microbiota composition to complement the available disease-specific markers.

## Introduction: Aging and Pathologies Share the Same Common Mechanisms

The longstanding question if old age is itself a disease has been addressed since ancient times, starting from the Roman playwright Terentius, who claimed “*senectus ipsa est morbus*” (old age itself is a disease), and Cicero who some decades later argued in *De Senectute*: “*pugnandum, tamquam contra morbum sic contra senectutem*” (we have to fight against aging, as we do against a disease). These quotations elegantly summarize a long-held view of aging and old age addressed by several scholars (see Appendix for further details). Notwithstanding, with the birth of modern medicine in the nineteenth century, this old tenet has been somehow put apart, as the main interest at that time was to define precise medical entities (diseases and syndromes) and their causes (infections, genetics, degenerative processes, inflammation, etc.). This process ended up in considering aging and diseases as separate phenomena that could eventually interact but that are essentially different in nature. In this review, we will reappraise and challenge the old tenet that aging and age-related diseases (ARDs) and geriatric syndromes (GSs) are separate entities, and we will suggest instead that both should be considered as parts of a continuum. To support this hypothesis, we will highlight that aging and ARDs/GSs share the same basic molecular and cellular mechanisms.

Aging is the predominant risk factor for most diseases and conditions that limit healthspan. Accordingly, interventions in animal models that end up in an extension of lifespan prevent or delay many chronic diseases. Why? For many years the explanation was that aging *per se* is a physiological condition, which favors the onset of many diseases. However, their relationship is likely much more complex, and a major reason is because they share the basic mechanisms. Assuming that aging and ARDs/GSs share the same mechanisms, which are commonalities and differences? In this review, we will argue that an integrated hypothesis, fitting most epidemiological and experimental data, is to consider ARDs/GSs as an acceleration of the aging process. The conceptualization of accelerated aging started from the observation of rare genetic disorders (1), including Hutchinson–Gilford progeria (2), mandibuloacral dysplasia (3), Werner’s syndrome (4), and aneuploidies such as Down syndrome (DS) (5). Here, we extend the concept of acceleration of aging to those members of the general population undergoing ARDs and GSs, in comparison with a small minority of people, such as centenarians, who reach extreme age largely avoiding or postponing most ARDs/GSs. This consideration is reinforced by the observation that among centenarians there are few subjects who never suffered of any overt ARDs. These exceptional individuals can be taken as a proof of principle that “healthy” aging and diseases can occur separately, as phenotypes at the extreme of a continuum, which is fueled by a common set of molecular and cellular mechanisms.

Which are the basic mechanisms shared by aging and ARDs/GSs? A group of international experts identified “seven pillars” which actually include adaptation to stress, loss of proteostasis, stem cell exhaustion, metabolism derangement, macromolecular damage, epigenetic modifications, and inflammation (6). Many chronic diseases and pathological conditions (listed in Table 1) are at least in part determined by (some of) these mechanisms, as it will be detailed in the next paragraphs, lending support to this hypothesis.

**Table 1:** Age-related pathologies and molecular relationship with aging.

Age-related pathology	Mechanisms shared with aging process	Markers	References/reviews
Alzheimer’s disease	Inflammation Oxidative stress Mitochondrial dysfunction Decreased proteasome activity Cellular senescence Gut microbiota alterations	IL-6, TNF-α, IL-1β, TGFβ, IL-12, IL-18, and INFγ 8-hydroxyguanosine, 8-hydroxy-2′-deoxyguanosine, oxidized proteins, and lipid peroxidation 20S core reduced activity Presence of senescent cells Activation of pro-inflammatory cytokines, increased intestinal permeability	(7) (8) (9) (10) (11) (12)

Cancer	Inflammation Cellular senescence	IL-6; presence of senescent cells	(13–16)

Chronic obstructive pulmonary disease	Telomere shortening Oxidative stress Cellular senescence Inflammation, inflammasome; activation of NLRP3 Activation of PI3 kinase–mTOR signal Dysregulated nutrients sensing; loss of proteostasis autophagy mitochondrial dysfunction Stem cell exhaustion	p21CIP1/WAF1, p16INK4a, β galactosidase activity, and senescence-associated secretory phenotype IL-1β, IL-6, IL-18, chemokines (CXCL8 and CCL2), metalloproteinases Stress markers such as Parkin and phosphatase and tensin homolog-induced protein kinase 1	(17, 18)

Maculopathy	Chronic retinal inflammation, dysregulation of autophagy, accumulation of oxidative stress-induced damage, protein aggregation, and lipofuscinogenesis	Heat shock proteins; Abs vs self-epitopes; and inflammasome activation	(19, 20)

Osteoarthritis	Cell disruption; cellular senescence; mitochondrial dysfunction and oxidative stress; and reduced autophagy; inflammation	HGMB1; HGMB2; and IL-8	(21)

Osteopenia/osteoporosis	Chronic inflammation	TNF-α; IL-6; CRP; and inflammatory markers	(22)

Parkinson’s disease	Inflammation Cellular senescence Gut microbiota alterations	Presence of inflammatory cells (astrocytes) and senescent cells Activation of pro-inflammatory cytokines, increased intestinal permeability, and alteration of the serotonin system	(23) (24) (25)

Periodontitis	Inflammation	*Porphyromonas gingivalis* express peptidylarginine deiminase generating citrullinated epitopes Pro-inflammatory cytokines	(26)

Rheumatoid arthritis	Cell death and chronic inflammation	Abs vs modified self-epitopes; HGMB1 Matrix metalloproteinases TNF-α; IL-1β; and IL-6	(27)

Sarcopenia	Inflammation and oxidative stress	Elevated levels of TNF-α, IL-6, IL-1, and CRP	(28–30)

Following this idea, the very difference between aging and diseases would relay on the rate/speed and intensity of aging cellular and molecular processes, combined with specific organ/systems genetic and lifestyle/habit predisposition. Thus, on the long run, all the functional domains undergo a physiological decline that eventually can lead to overt clinical diseases, favored by organ/system-specific genetic and environmental factors. This progressive path generates a continuum between the healthy juvenile status and the impaired unhealthy elderly one. Accordingly, all major ARDs/GSs are characterized by a long subclinical incubation period, where the diagnostic signs of diseases are largely unobservable due to the high operational redundancy of biological systems. This redundancy, together with the progressive capacity of cells and systems to adapt (“remodeling theory of aging”) (31, 32) is capable to buffer the progressive accumulation of molecular damages, thus hampering the availability of objective early diagnostic signs/tools. As an extreme example in neurodegenerative diseases such as Parkinson’s disease (PD), it is possible to ascertain advanced anatomopathological alterations in the absence of any specific clinical symptoms in patients died of other diseases.

Accordingly, aging on one side and ARDs/GSs on the other have to be considered different trajectories of the same process but with a different rate depending on diverse genetic background and lifestyle (33–35). Some considerations can help the reasoning on this topic: (i) aging has not been selected during evolution, and no gerontogene has been identified so far, i.e., no gene has been apparently selected with the precise purpose to trigger/cause the aging phenomenon, thus leaving a large space for stochasticity (36); (ii) genetics and environment interact with each other to determine the eventual phenotype. These two considerations can explain (a large part of) the heterogeneity of phenotypes observed in aged persons. Actually, the primary aim of a gene (or group of genes) is always devoted to increase the survival or reproductive fitness of the organism, and aging could be an unpredicted byproduct of its basic function. Following this idea, some years ago Mikhail Blagosklonny and Michael Hall proposed that aging could be conceptualized as a sort of dysregulated continuation of the normal developmental process and related cellular “programs,” with particular emphasis on mTOR-driven growth (37, 38). According to this theory, overactivation of signal transduction pathways and exacerbation of normal cellular functions such as growth, leading to alteration of homeostasis, malfunction, and organ damage are likely the driving forces of the aging process including the onset of ARDs. This theory complements the “inflammaging” theory of aging (39). Inflammation is among the aforementioned “seven pillars,” and inflammaging is defined as the chronic, low-grade (subclinical) and sterile inflammation that is observed in old persons. It is caused by increased stimulation of innate immune system by “non-self” (persistent infections), “self” (cell debris, nucleic acids, glycated proteins, etc.), and “quasi-self” [gut microbiome (GM)] components of our body as a meta-organism, and by accumulation of senescent cells characterized by a pro-inflammatory secretory profile (40, 41). Thus, both the overactivation and inflammaging theories agree that programs selected for development and survival (inflammation) can turn detrimental when continue to be active unabated for a period time longer than that predicted by evolution. The same can apply for other programs of the abovementioned “seven pillars.”

## Age-Related Pathologies

In this paragraph, we will discuss the involvement of some molecular mechanisms known to cause aging in a number of ARDs/GSs, in particular frailty and sarcopenia, chronic obstructive pulmonary disease (COPD), cancer, and Alzheimer and Parkinson diseases. We will also discuss the manifestations of premature senescence of a genetic syndrome, such as DS, which are present also in normal aging but occur much earlier at the level of immune and nervous system in DS. Cardiovascular diseases and type 2 diabetes are also very important pathologies that affect millions of patients and do share molecular mechanisms with aging, including inflammation and oxidative stress, but for reasons of space limitations, a detailed discussion of these pathologies will be skipped out.

### Frailty Syndrome

Typical GSs include frailty, mild cognitive impairment, and metabolic syndrome. In particular, frailty is described as a multidimensional syndrome of the elderly characterized by a loss of physiological reserves, poor response to, and recovery from (even mild) stress. This condition leads to an increased vulnerability to a wide range of adverse health outcomes and is associated with increased morbidity and mortality. However, frailty is peculiar as it displays a wide spectrum of phenotypes depending on the criteria that are considered for its definition, as well as the age range of the subjects studied. To this regard, both clinicians and researchers are becoming more and more aware of the considerable ambiguity around the concept of frailty. Conflicting ideas have proliferated on the definition of frailty, what criteria should be used for its recognition, and its molecular relationships with aging, disability, and chronic diseases (42).

In the 2001, Fried et al. elaborated five criteria to define frailty, i.e., unintentional weight loss, poor hand grip, slow speed gate, feelings of exhaustion and low physical activity. Persons (usually older than 65 years) meeting three or more out of these five criteria are classified as frail and have an increased risk of incident falls, worsening mobility or ADL disability, hospitalization, and death in the following 3 years. Intermediate frailty status or pre-frail condition, as indicated by the presence of one or two criteria, showed intermediate risk of these outcomes (43). During the last decade, several frailty-rating scales have been developed to detect and screen the level of frailty, such as the Frailty Risk Index (44–46). An impressive amount of literature has been published suggesting that a complex network of clinical signs produce a large spectrum of frailty conditions and phenotypes (47) with different risk index of mortality after 3–5 years of follow-up. Surprisingly, the condition of frailty may be reverted and subjects can return to a non-frail condition, for example, when specific pathologies are cured, or personalized interventions in terms of physical exercise, with or without nutrition supplementation, are applied (48, 49).

This quite peculiar condition deserves some assumptions, such as that frail is an epiphenomenon and “etiology” may be quite different in the population, also depending on the possible overlapping with sarcopenia, i.e., the age-related loss of muscle mass and strength that will be discussed later. Actually, many signs of frailty are related to sarcopenia, and if both conditions are present in the same individual, they favor the state of vulnerability, increasing the risk of negative health outcomes. Nevertheless, a low number of studies have assessed the coexistence of these two entities in the same cohort of older people.

Recently, the Toledo Study of Healthy Aging (a study of 65+ community-dwelling elderly) including 1,611 participants with frailty and sarcopenia assessments indicated that the prevalence of frailty (assessed by Fried’s criteria) among those with sarcopenia was from 8.2 up to 15.7%, depending on the different criteria for sarcopenia assessment. Moreover, among frail people, the prevalence of sarcopenia was from 40.27 up to 72.2%, according to the used criteria. Sarcopenia showed a low sensitivity but high specificity for the diagnosis of frailty thus suggesting that frailty and sarcopenia are distinct but interrelated conditions (50).

Furthermore, the molecular mechanisms underpinning frailty syndrome are still not completely clarified even if many data suggest a tight relationship with inflammatory status and immunosenescence which are also shared in sarcopenia (30), as it will described below. Likely, both frailty and sarcopenia contribute to further development of morbidities. Importantly, the role of inflammaging to the frailty syndrome onset is still an open question (51), and further studies are needed to clarify the causality between chronic low-grade inflammation and development of frailty, as well as the conditions/treatments that make possible the reversibility of the frailty status. It is important to note that Fried and Ferrucci (52) were the first to elaborate the concept of frailty as a syndrome of “accelerated aging” and to note that clinical frailty is associated with the presence of multiple chronic diseases. In turn, the risk to become frail increases with the number of such diseases present, thus reinforcing the idea of a continuum between health, diseases, and comorbidity. To this regard, a multidimensional approach allows a more robust interpretation of the various relationships among the pro- and anti-inflammatory markers and inflammaging (53), and likely an important contribution could be obtained by introducing also frailty risk and mortality indexes in a context of a complex dynamical network (54) to better disentangle those clustering factors that may accelerate aging.

### Sarcopenia

One of the most pervasive and macroscopic phenomena occurring with aging is the progressive decline of skeletal muscle mass, strength, and function, leading to a condition indicated as sarcopenia. Sarcopenia is associated with a reduced quality of life in older adults, and it is considered as a key risk factor for negative health outcomes associated with disability, frailty, loss of independence, morbidity, and mortality (55, 56). Several factors are involved in the pathophysiology of sarcopenia; however, its etiology is still unclear. The more recent evidence suggests that the onset and progression of sarcopenia depend on a combination of mechanisms that alter the normal physiology of skeletal muscle, some of them being considered also as key driver of the aging process. Among the mechanisms that participate to the pathogenesis of sarcopenia there are endocrine changes, loss of regenerative capacity, muscle fiber denervation, increased deposition of intermuscular and intramuscular fat, mitochondrial dysfunction, oxidative stress, and inflammation (57, 58). These two latter mechanisms in particular are involved also in the aging process. Loss of regenerative capacity of satellite cells (the stem cells of the muscle) is another feature shared with aging. However, this mechanism, although important *in vivo*, has been put under scrutiny and will not be further discussed here. In fact, satellite cells from old muscles display a proliferative capacity similar to those derived from young muscles, if cultured in an appropriate medium enriched with plasma from young donors (59, 60), therefore casting some doubts on the fact that satellite cells from old muscle are actually defective or exhausted. On the contrary, these data suggest that stemness as well as other features of satellite cells are strongly dependent on the environmental context (namely but not exclusively soluble factors) and are therefore cell independent.

Actually, emerging epidemiological and molecular studies indicate that immunosenescence and inflammaging strongly contribute to the pathophysiology of sarcopenia (30, 61). The age-related changes in the cells of the innate immune system indirectly contribute to sarcopenia by an increase of systemic inflammation. In physiological conditions, in response to damage, neutrophils migrate in skeletal muscle, followed by M1 macrophages that lead to muscle inflammation. This early phase is followed by infiltration of M2 macrophages that produce soluble factors that repair the muscle injury and promote regeneration (62). With aging, the activity of neutrophils decreases, especially in terms of migration capacity. It has been hypothesized that, once in the muscle, neutrophils with impaired migration capacity can contribute to increased inflammation (30). The incomplete muscle recovery is associated with an increase of pro-inflammatory cytokines and a prolonged inflammatory response to muscle injury that causes muscle atrophy and weakness (28).

Regarding systemic inflammation a possible involvement in sarcopenia has been proposed and depends upon the degree of intensity of inflammation. A comparative analysis of skeletal muscle alteration at different ages from four species, i.e., mice, rats, rhesus monkeys, and humans, revealed not only a conserved age-dependent decrease in mitochondrial content, and a reduction in oxidative phosphorylation complexes in monkeys and humans but also a human-specific age-related increase of phosphorylated NF-κB (63). Actually, a moderate inflammation is beneficial and fundamental to activate a response to a stress, but when the inflammation becomes chronic and more elevated, the response to muscle injury turns detrimental. In other words, a mild level of systemic inflammation present in physiological aging may not affect the loss of muscle mass or strength, but only the metabolic quality of skeletal muscle; conversely a more severe systemic inflammation (often accompanied by a local inflammation) present in a condition of accelerated aging, contributes to the loss of muscle mass and strength and the progression of sarcopenia (30). Although the molecular mechanisms associated with inflammaging and the loss of skeletal muscle mass are not yet totally understood, studies revealed that inflammaging contributes to the genesis of sarcopenia by affecting the balance between muscle anabolic and catabolic processes (64). In particular, elevated levels of TNF-α, IL-6, IL-1, and CRP favor muscle protein breakdown and inhibit protein synthesis through the activation of NF-κB and ubiquitin–proteasome pathway. This shift toward catabolic process then culminates in myofiber proteolysis, atrophy and loss in regenerative ability that leads to skeletal muscle functional decline (29, 30). Emerging evidence indicates that the progression of sarcopenia is also amplified by a self-sustaining loop between immunosenescence, inflammaging, and oxidative stress (58, 61). There is in fact a close interconnection and/or overlapping between the molecular pathways of inflammation and those of oxidative stress in the generation of reactive oxygen species (ROS). These species have pathological consequences for the health of human body not only associated with the development of sarcopenia but also a number of other ARDs, including typical age-related endocrine dysfunctions such as decreased pancreatic β-cell function and thyroid autoimmunity, among others (65). An uncontrolled accumulation of oxidative stress and inflammation may act as a bridge between normal aging and accelerated aging. In conditions of accelerated aging, muscle weakness is often accompanied by other pathophysiological features, such as low bone density and increased fat mass, thus leading to osteoporosis and obesity. All these disorders have been recently indicated with the term “osteosarcopenic obesity” (66), and, as mentioned earlier, they can be listed among the determinants of frailty (30, 67).

### Chronic Obstructive Pulmonary Disease

Aging is one of major risk factors for many chronic inflammatory diseases, e.g., diabetes, CVD, atherosclerosis, dementia, cancer, and others including COPD, and can impact differently on organs and tissues affecting their functions and structure (68).

Aging of the lung is characterized by reduction of function, pulmonary inflammation, increased gas trapping, loss of lung elastic recoil and enlargement of the distal air space. These pathological signs are slowly progressive and are also pathognomonic of COPD. In fact, the overall increase in COPD is probably related to the aging of the population, as this disease predominantly affects the elderly, with the peak of prevalence at about 65 years (69, 70). COPD is an obstructive lung disease characterized by long-term breathing problems, poor airflow, and destruction of the lung parenchyma (emphysema) (71). The main cause of COPD in industrialized countries is smoking but is also present in underdeveloped countries as a result of exposure to household air pollution, poor nutrition, and damp housing conditions (72–74). The slowly progressive airway obstruction of COPD and in particular the emphysema could represent an acceleration of the normal decline of lung function with age (75, 76).

Recent and extensive studies (18, 77) have pointed out that in COPD are present many of the hallmark of aging, e.g., telomere shortening, activation of PI3 kinase–mTOR signaling, altered autophagy, mitochondrial dysfunction, stem cell exhaustion, as well as a low-grade inflammation and cellular and immune senescence.

Telomere attrition leading to cellular senescence (replicative senescence) or cell death, have been described in leukocytes from patients with COPD in comparison with control subjects in any age range (78). Moreover, parenchymal lung cells of emphysematous patients display shorter telomeres associated with cell senescence and inflammation (79, 80). In a meta-analysis of 14 studies, a significant negative association between telomere length and COPD has been observed (81). This telomere shortening in COPD could be due to an augmented oxidative stress from cigarette smoke that activates p21, leading to cellular senescence and increased release of pro-inflammatory cytokines (79). Cell senescence in COPD is evident by the enhanced expression of senescence markers such as p21CIP1/WAF1, p16INK4a, and senescence-associated β-galactosidase activity in lung cells (82). Lung macrophages from COPD patients can also express senescence markers (18). Furthermore, in COPD there is an increased expression of components of the secretory profile of senescent cells, defined as senescence-associated secretory phenotype (SASP), including pro-inflammatory cytokines (IL-1 and IL-6), chemokines (CXCL8 and CCL2), and matrix metalloproteinase (MMP) 9 (18). As mentioned, SASP, in association with immunosenescence, is a key determinant of inflammaging that have a negative impact in the neighboring lung tissue and, as discussed, probably also in the whole organism (17).

The immunosenescence of both innate and adaptive immune cells and the consequent inflammaging might play a role in COPD development and progression. Recently, it has been demonstrated in aged mice exposed to chronic cigarette smoke, that activation of immune system and inflammaging contribute to the accelerated pathogenesis of emphysema, the increased chronic lung tissue inflammation due to the increased production of inflammatory mediators and this promotes the onset of COPD (83).

The mTOR pathway has an important role in cellular senescence and aging. In fact, an inhibition of this pathway extends the lifespan of many species (84). The activation of PI3 kinase–mTOR signaling pathway has been demonstrated in epithelial cells from the lungs of patients with COPD. The activation of the IGF-1/AKT/mTOR pathway suppresses autophagy, but it also counteracts activation of FOXO transcription factors, which are central regulators of metabolism, cell-cycle progression and programmed cell death (85). A diminished expression of FOXO3 protein has been demonstrated in the lungs of smokers and patients with COPD suggesting that dysregulated nutrient sensing, together with loss of proteostasis, may contribute to the pathogenesis of COPD (85, 86). Two central mechanisms are involved in proteostasis to degrade and remove the misfolded or damaged proteins, i.e., autophagy–lysosome system and the ubiquitin–proteasome system. The impairment of these pathways characterizes numerous ARDs but also the aging process itself (87, 88). A large amount of data indicate that the mechanisms involved in homeostasis and proteostasis collapse with advancing age, favoring the accumulation of the unfolded, misfolded, or aggregated proteins (89). The decline of ubiquitin–proteasome system during aging may be due to various alterations including decreased expression of proteasome subunits and insufficient or inappropriate assembly; reduction of proteasome function due to decreased ATP availability from mitochondrial malfunction. An increase of inducible subunits has been demonstrated as consequence of the abovementioned alterations in many tissue and organs (e.g., the skeletal muscle). This induction could be a compensatory mechanism altering the balance between constitutive proteasomes and immunoproteasomes and an effect of inflammaging (90).

A decline in proteasome activities has also been reported in human senescent fibroblasts (91). Conversely, the fibroblasts from centenarians, a group of individuals who have gone through the aging process successfully because they maintain their good mental and physical shape, show levels of proteasome activities, oxidized proteins, and RNA and protein expression of several proteasome subunits similar to the levels found in cultures obtained from young donor. Consequently, maintenance of proteasome function in centenarians has been suggested to be an important factor for their successful aging (92). Collapse of the mechanisms that lead to failure of proteostasis may have detrimental consequences for organisms. For example, failure of the proteasomal system has been linked to several pathologies, including neurodegenerative diseases (e.g., Alzheimer’s; Parkinson’s; and Huntington’s), cardiovascular diseases (e.g., atherosclerosis), immune system-associated diseases [e.g., rheumatoid arthritis (RA)], skin aging, cancer, and COPD, among others (93). In COPD, the oxidative stress induced by cigarette smoke can alter the proteins such as histone deacetylases contributing to their inefficient degradation by proteasome system or by autophagy (94, 95). Proteasome activity is decreased in patients with COPD and correlates inversely with the loss of lung function (96). Moreover, alveolar macrophages from cigarette smokers showed defective autophagy that could contribute to the accumulation of damaged proteins, abnormal mitochondrial function, and defective clearance of bacteria (97). There is evidence of increased markers of autophagy in lung tissue from patients with emphysema, suggesting that autophagy may be contributory to the apoptosis and alveolar destruction in emphysema (96). As abovementioned autophagy is also impaired through the activation of phosphoinositide 3-kinase–mTOR signaling in COPD (98) and may contribute to defective phagocytosis of bacteria in COPD (99).

Mitochondrial dysfunction is also present in COPD. In particular, an increased mitochondrial ROS production and a reduced number of mitochondria are typical features of the disease (100). The airway epithelial cells from smokers display an altered mitochondrial structure and function (101), and actually markers of mitochondrial stress such as increased expression of Parkin, phosphatase, and tensin homolog–induced protein kinase 1 are present in epithelial cells from patients with COPD (102). These changes in epithelial cells are accompanied by an augment in pro-inflammatory cytokines secretion such as IL-1β, IL-6, and CXCL8 (101). Mitochondrial alterations and ROS production can induce the NLRP3 inflammasome, which stimulates IL-1β and IL-18 secretion in chronic inflammatory diseases. The transcription factor peroxisome proliferator-activated receptor-γ coactivator (PGC)-1α is a critical regulator of mitochondrial biogenesis and the generation of mitochondrial ROS. It is increased in epithelial cells of mild COPD patients but progressively reduced with increasing COPD severity (103).

Finally, stem cell exhaustion, typical of aging process, is also present in COPD. The basal progenitor cells required for air way epithelial differentiation actually display a reduced regenerative capacity in COPD patients (104).

### Cancer

Many types of cancer are essentially ARDs, as their frequency dramatically increases with age, and age represents the single most powerful risk factor for cancer to occur. This phenomenon is likely not linked to a decreased efficiency of DNA mutation checkpoint and repair. Conversely, a growing amount of evidence suggests that the increasing number of transforming mutations occurring in old subjects is fostered by a much more permissive environment that allows DNA damage to occur and, probably most important, allows transformed cells to progress into malignancy and metastatization. The main feature of such a permissive environment is likely the presence of an elevated level of pro-inflammatory stimuli, either related to the immune response to cancer or independent from it. Actually multiple lines of evidence indicate that immune inflammatory cells can actively promote tumor growth, as such cells are capable of fostering angiogenesis, cancer cell proliferation, and invasiveness (16). Therefore, a positive response aimed at counteracting cancer has the paradoxical effect of promoting tumor growth, invasion, and metastasis (105–108). Importantly, inflammation is in some cases evident at the earliest stages of neoplastic progression and is demonstrably capable of fostering the development of incipient neoplasias into full-blown cancers (107, 109), as inflammatory cells can release ROS that are actively mutagenic for nearby cancer cells, accelerating their genetic evolution toward heightened malignancy (106). Stressed or necrotic cells can be the source of molecules that can attract inflammatory cells leading to the abovementioned promoting effects on the tumor, as seen, for example, in melanoma, where exposure to UV light leads to the release of HMGB1 protein from keratinocytes, that in turn attracts and activates neutrophils and induces the production of angiogenetic factors (110). The same effects can be obtained even in the absence of an infiltration of inflammatory cells, granted that other cells can sustain the production of the same array of pro-inflammatory mediators. This is the case when senescent cells accumulate in a tissue. Cell senescence is an effective mechanism to halt neoplastic transformation, as cells with damaged DNA can enter cell senescence and stop proliferating. However, as mentioned, senescent cells are characterized by a pro-inflammatory secretory phenotype (SASP) (111) that includes metalloproteinases and angiogenetic factors. Many of these factors can contribute to the acquiring of malignant and metastatic features of cancer cells (112). Therefore, the occurrence of an antineoplastic mechanism can paradoxically end up in fostering the neoplastic transformation of premalignant cells through SASP (13, 14). Actually, it is known that aging is characterized by accumulation of senescent cells, due to either inefficient clearance or increased number of cells undergoing this process, and, accordingly, SASP is considered a main driver of inflammaging. SASP, in turn, can ignite DNA damage response and synthesis of pro-inflammatory cytokines in surrounding cells in a self-amplifying loop, leading to the proposal that inflammaging can be a substantial driver of the increase in cancer incidence and progression observed in aged people (113).

The phenomenon of inflammaging (at the level of stem cell niche) can be therefore a risk factor for cancer development and, since inflammaging increases with age, this would account for association between cancer and old age. This can be exemplified by the case of myeloproliferative neoplasms (MPNs), which are acquired age-associated clonal disorders of the hematopoietic stem/progenitor cells (HSPCs). MPNs are characterized by a state of chronic inflammation due to the continuous release of inflammatory products from *in vivo* activated leukocytes. This state of chronic inflammation (or inflammaging) affects both the malignant HSPCs and the non-malignant/malignant microenvironment, likely being the main contributor in MPNs initiation/clonal evolution (114, 115). This inflammatory microenvironment is a key factor in MPNs pathogenesis, since strong evidences suggest that stromal cells are primed by the malignant hematopoietic clone, which, in turn, conditions the stroma to create a favorable microenvironment that nurtures and protects the malignant cells (116).

Among the classical component of inflammaging, IL-6 occupies a prominent place. It has been demonstrated that IL-6 drives the progression toward the acquisition of a malignant phenotype of cancer cells (15) and that the blockade of IL-6 signaling has strong effects *in vivo* on tumor progression, interfering broadly with tumor-supportive stromal functions, including angiogenesis, fibroblast infiltration, and myeloid suppressor cell recruitment in both the tumor and premetastatic niche (117).

As a whole, it is widely accepted that inflammation and cancer are strictly connected and that inflammation is involved in cancer onset and progression. Inflammaging seems to be an almost universal feature of human aging, so it can be hypothesized that if a subject could live long enough, the effect of inflammaging on his/her probability to get cancer would become very important. Similarly, it can be reasoned that a person who got cancer at 60 years of age is comparable (as far as inflammaging is concerned) to a much older person, thus it could be speculated that cancer is to a certain extent a consequence of an accelerated aging process. To further support this hypothesis, it is known that many syndromes of premature, accelerated aging like Werner syndrome and ataxia telangiectasia are also characterized by increased frequency of malignancies (118, 119). On the other side, centenarians (who can be considered to be biologically younger than their chronological age) rarely die by cancer (120). Of course many factors concur in malignant transformation other than inflammation; however, this fascinating hypothesis certainly deserves further investigations.

### Neurodegenerative Diseases

Alzheimer’s disease (AD) and PD are the most common neurodegenerative diseases in the world (121). These diseases are age-associated and most often have a long prodromic phase preceding the clinical manifestation with a subsequent stage of progression leading to signs of dementia with similar symptoms such as memory impairment, orientation problems, and difficulties in performing service functions among others. AD and PD are referred to as “protein misfolding” diseases because deposits of improperly folded modified proteins are detected in specific areas of the brain (122–124). In the case of AD, these deposits contain β-amyloid proteins and hyperphosphorylated tau protein (tau-P), which, respectively, form extracellular plaques and intracellular fibrillar tangles (125). In the case of PD, the deposits—called Lewy bodies—are formed by the accumulation of α-synuclein protein in dopaminergic neurons mainly of the *substantia nigra*, as well as in other regions of the brain (126). In both AD and PD, neurodegeneration processes are generally accompanied by neuroinflammation (127).

#### Alzheimer’s Disease

The clear diagnosis of AD is made only postmortem, and no effective disease-modifying therapy exists at the moment (128). On living patients, AD is diagnosed by a combination of cognitive tests and neurobiological markers [brain imaging, decreased amyloid beta Abeta42 (Ab) level and/or increased total and hyperphosphorylated tau-P in cerebral spinal fluid] (129). These tissue changes precede the onset of clinical signs by several years, implying that AD neuropathological lesions may be found in a subset of cognitively normal elderly persons (130). This suggests that (i) although senile amyloid beta (Abeta) plaques play a role in the AD dementia, the scenario is more complex and other (major) drivers are also involved; (ii) there is a *continuum* between neurodegenerative AD dementia and the dementia-free brain aging. The limits of the current conceptualization on AD pathogenesis and of the amyloid cascade hypothesis are well summarized by two Nature Neuroscience papers released in 2015 (131, 132). In fact, many other potentially harmful phenomena take place in AD pathogenesis, some of them being shared with the aging process, such as oxidative stress, mitochondrial dysfunction, neuroinflammation, decrease in proteasome activity (10) and deregulation of basic mechanisms of cell functioning (autophagy and DNA damage response). In many cases, these phenomena are not immediately connected to Abeta deposition and neurofibrillary changes. In particular, neuroinflammation in AD involves not only resident cells (microglia, astrocytes, and neurons) but also cells and soluble factors of the peripheral immune system that can enter into the brain (133). To this regard, inflammaging can stimulate the development of neuroinflammation and neurodegeneration (134, 135). This effect is due to soluble mediators that can enter the blood–brain barrier, essentially cytokines, whose network can be deranged in AD (136), therefore the assessment of peripheral inflammatory markers should be considered in the monitoring of the efficacy of therapeutic approaches. A meta-analysis demonstrated that increased serum levels of IL-6, TNF-α, IL-1β, TGFβ, IL-12, IL-18, and IFNγ characterize AD (7). Interestingly, IL-6 is capable of entering the blood–brain barrier and has a role in memory consolidation (137). The pro-inflammatory cytokines IL-1β and TNF-α exert variable (inhibiting or supporting) synapse-specific effects on long-term potentiation maintenance (138). It was also shown that IL-1β and TNF-α in combination with IFNγ can exacerbate the pathology in AD due to alterations of the β-amyloid precursor protein (βAPP) metabolism resulting in triggering the production of β-amyloid peptides (139, 140).

The balance of antioxidant and oxidant system activity is deranged in cells affected by AD. Elevated levels of oxidative stress markers are also present in mitochondria isolated from peripheral lymphocytes of AD patients (141). Mitochondrial DNA (mtDNA) inherited mutations have also been associated with AD onset (142). AD patients are characterized by significant increases in blood cells of markers of oxidative stress for both RNA (8-hydroxyguanosine) (8) and DNA (8-hydroxy-2′-deoxyguanosine), together with a considerable decrease in antioxidant defense (9, 143, 144). Moreover, high levels of oxidized proteins and of products of lipid peroxidation are also found. In particular, a significant increase in the degree of lipoprotein oxidation was observed in the peripheral blood of AD patients (145). Neutrophils are the main source of ROS production in the sites of inflammation. A possible participation of neutrophils in the development of AD has been demonstrated (146). Oxidative stress in neurons it is also able to produce a DNA damage response that in turn leads to apoptosis or cellular senescence (11). A potential contributor to age-related inflammation in the brain can then be cellular senescence, likely occurring in replication-competent glial cells. Recent studies from several laboratories suggest that senescent cells are detectable in the mammalian brain, where they could contribute to neurodegenerative processes with their pro-inflammatory SASP and/or disrupting cell–cell contacts needed for the structural and functional neuron–glial interaction that maintains neuronal ionic and metabolic homeostasis (147, 148). Senescent markers were recently reported to be present in astrocytes of autopsied human brain tissue; both p16INK4a and the SASP factor MMP3 increased significantly with age and were even higher in affected cortical brain tissues from AD patients compared with age-matched non-demented controls (149).

Gut and brain are deeply interconnected through the gut–brain axis (150). Inputs from the CNS can modify gut functions, while inputs from gut to CNS can modulate specific symptoms (151). Alterations of these bidirectional communications may contribute to neuroinflammation and the pathogenesis of CNS disorders (152). In particular, alterations of GM can activate pro-inflammatory cytokines and increase intestinal permeability, leading to the development of insulin resistance, which has also been associated with AD (12). In addition, bacteria of GM are known to excrete immunogenic mixtures of amyloids, lipopolysaccharides, and other microbial exudates into their surrounding environment (153, 154). Bacterial amyloids might activate signaling pathways known to play a role in neurodegeneration and AD pathogenesis, while GM might enhance inflammatory responses to cerebral accumulation of Ab (155). It is also interesting to mention that beside gut microbiota, the oral microbiota is involved in several pathologies including AD. Aging may favor the proliferation of anaerobes in the mouth eliciting a robust TNF-α response by the oral epithelium (156). In AD brains, a sevenfold higher presence of anaerobe oral bacteria compared with cognitively normal controls has been found (157). The causal link between bacteria and AD-like neurodegeneration has been further illustrated in a mouse model (158).

#### Parkinson’s Disease

Parkinson’s disease is caused by the selective loss of neurons of the substantia nigra due to improper accumulation of α-synuclein protein leading to motor alterations. Despite this apparently very specific cause, PD actually shares some feature with normal aging and could be considered a segmental accelerated aging that affects specific neurons in the brain and in many other anatomical sites. First of all, features of PD are found also in elderly without clinical sign of PD (159). A study on 2,500 old persons annually assessed for Parkinsonism showed that 744 of these subjects deceased without diagnosed PD (mean age at death: 88.5 years): (i) about one-third of cases had mild or more severe nigral neuronal loss; (ii) about 17% had Lewy bodies; and (iii) 10% of the brains showed both nigral neuronal loss and Lewy bodies (160). Thus, also in this condition there is an apparent continuum between physiological aging and neurodegenerative age-related motor disorders.

Recent data indicate that aging and PD share basic characteristics such as accumulation of senescent cells, inflammation, and propagation phenomena. It has been reported that senescent and inflammatory cells (astrocytes) are present in the brain of PD patients (23) and a “transmission hypothesis” has been proposed regarding the pathogenesis of “PD as a prion disease” (161) where intercellular transmission of pathological protein aggregates (α-synuclein) occurs, causing a prion-like spreading of neuronal damage and neuroinflammation (162, 163). Aggregated α-synuclein, released by neuronal degeneration, acts as an endogenous trigger inducing a strong inflammatory response in PD (164). Similar propagation phenomena have been described for beta-amyloid and Alzheimer’s diseases (165).

Increasing evidence suggests that PD should be included on the growing list of diseases associated with vitamin D insufficiency and that we should routinely monitor vitamin D levels in patients with PD (166). One of the most advanced and appealing hypotheses is that environmental stressors may contribute to age-related neurodegeneration by favoring cell senescence of glia, thus creating a chronically inflamed milieu in the brain (167). From this point of view it is important to note that a bidirectional axis between the brain and the GM does exist, and, importantly, GM is involved in the production of various neurotransmitters (serotonin, dopamine, noradrenaline, and GABA), and in the modulation of various behavioral and CNS functions (168, 169). Recent studies showed that PD is associated with gut dysbiosis (24, 170), the fecal concentration of short-chain fatty acids is significantly reduced in PD patients compared with controls, and this reduction could impact on CNS alterations and contribute to gastrointestinal dysmobility in PD (171). In a mouse model of PD, it has been demonstrated that GM is key player in motor deficits and microglia activation (172).

On the basis of the profound even if still unclear relationship between aging and PD, these data on PD microbiome should be interpreted on the background of the changes that occur in the GM during healthy aging. It has been recently showed that the GM undergoes profound changes with age (173), which likely contribute to inflammaging (174) and can have profound effects on the brain, owing to the increased abundance with age of bacteria involved in the tryptophan metabolism pathway (175), in agreement with the reduction of tryptophan (a precursor of serotonin) found in the serum of centenarians (32, 176). Accumulating evidence shows that the age-related dysbiosis is involved in the neurological decline and promotes inflammaging (177) that play a pivotal role in both the physiological and the pathological cognitive decline (25). The GM contributes to the regulation of the brain function modulating the metabolism of tryptophan, an essential amino acid derived from the diet that is able to cross the blood–brain barrier contributing to the synthesis of the serotonin in the central nervous system (25). The age-related changes are more evident in the amygdala, hippocampus, and frontal cortex. The function of these brain areas is strongly dependent from the serotonergic neurotransmission and thus involving the changes in the tryptophan GM-dependent metabolism. Alterations in the serotonin system could represent the common denominator of the alterations of the sleep, mood, and sexual conduction often observed in elderly as well as of other modifications such as diabetes and cardiovascular diseases (25). Tryptophan is also metabolized *via* the kynurenine pathway (KP), which can lead to the production of nicotinamide adenosine dinucleotide (NAD+) (168), as well as quinolic and kynurenic acid. These latter compounds are neuroactive metabolites that act on *N*-methyl-d-aspartate (NMDA) and alpha 7 nicotinic acetylcholine receptors in CNS and ENS. In the CNS, kynurenic acid has been long viewed as neuroprotective, while quinolinic acid is primarily considered an excitotoxic NMDA receptor agonist (178).

Alterations of the KP have been assessed in PD (as well as other neurodegenerative diseases). PD patients have higher l-kynurenine/tryptophan ratios in serum and CSF as compared with controls, suggesting upregulated activity of enzymes involved in catabolizing tryptophan to kynurenine [i.e., indoleamine-2,3-di-oxygenase and tryptophan 2,3-dioxygenase]. Levels of 3-hydroxykynurenine have also been found to be increased in the putamen, prefrontal cortex, and substantia nigra pars compacta in PD patients (179).

Despite the fact that periodontal diseases resulted associated with PD, few data are present on the role of oral microbiota in PD. A recent paper showed that oral microbiota of PD patients differs from those of control subjects as assessed through beta diversity and differential abundance analyses. Differences were also detected between sexes, with a higher abundance of taxa that include opportunistic oral pathogens in males (180).

### Other Pathologies: RA, Osteoarthritis (OA), Osteopenia, and Macular Degeneration

It is well known that chronic inflammatory (or autoimmune) diseases, such as RA, psoriasis, ankylosing spondylitis, OA, systemic lupus erythematosus, multiple sclerosis, inflammatory bowel diseases, and pemphigus vulgaris among others, share an inflammatory component highly depending on immune system activation, self-epitopes, environment-associated variables, and genetic makeup. In this review, we focus on osteoarticular pathologies and macular degeneration since an impressive amount of data is recently emerged. These data converge on the chronic inflammatory process, which drives the evolution of the disease as a continuum. Among osteoarticular pathologies, elderly onset RA usually develops in persons older than 60–65 years of age. Main actors involved in the RA development are activated T/B cells, macrophages, and fibroblasts producing pro-inflammatory cytokines that play a key role in synovitis and tissue destruction. In particular, TNF-α and IL-1β are two of the main cytokines that enhance synovial proliferation and stimulate secretion of MMPs, other inflammatory cytokines, and adhesion molecules (181). Recently, the role of HGMB1, released from dead cells, has been focused as a mediator of local and systemic inflammation being able to bind to RAGE, TLR2, and TLR4, to activate NF-κB and to induce the expression of the downstream cytokines including IL-6 (27, 182). Importantly, both TNF-α and IL-1β are included in the cytokine profile characterizing inflammaging (183), and HGMB1 is hypothesized to be one of molecules fueling this process (40) suggesting that inflammaging can be an additional cofactor involved in the pathogenesis of RA.

Furthermore, scientists have also pointed out the tight molecular relationship between periodontitis and RA pathogenesis consisting in an increased numbers of citrullinated epitopes, likely produced by specific human bacteria (*Porphyromonas gingivalis*) able to express peptidylarginine deiminase, an enzyme that generates citrullinated epitopes that are recognized by anti-citrullinated protein antibodies. Both diseases involve chronic inflammation fueled by pro-inflammatory cytokines, connective tissue breakdown and bone erosion as reviewed very recently (26). Thus, other mechanisms, such as the release of damage-associated molecular patterns from neutrophils may accelerate local and systemic inflammation as well as occur during aging (40), making evident the network structure of the involved molecules/markers and propagation mechanisms.

Aging is also the major risk factor for OA, which begins with disruption of the superficial zone of cartilage without any involvement of immune system, leading to progressive cartilage erosion and bone remodeling, causing disability and decreasing the quality of life. HMGB2 expression is uniquely restricted to cells in the superficial zone of normal mature human articular cartilage, and importantly, joint aging in humans and mice leads to the loss of HMGB2 expression while HMGB1 expression results increased in human OA-affected cartilage compared with normal cartilage (184). The contribution of HMGB1 to localized or systemic inflammation is mediated by innate immunity receptors, as described previously, leading to the increase of inflammatory status due to the production of chemokines and in particular IL-8 (185). Furthermore, many molecular and cellular mechanisms involved in inflammaging, such as cellular senescence; mitochondrial dysfunction and oxidative stress, dysfunction in energy metabolism associated with reduced autophagy and alterations in cell signaling were recently highlighted as processes contributing also to the development of OA (21). These processes promote a pro-inflammatory, catabolic state accompanied by increased susceptibility to cell death that together lead to increased joint tissue destruction and defective repair of damaged matrix.

Osteopenia is a condition not only highly associated with the aging process but also to different acute inflammatory diseases, leading to episodic bone reabsorption. Long-term solicitation of this process (22) might induce low bone mass and lately osteoporosis. Indeed, bone loss is typical in chronic inflammatory diseases (186–192) and other conditions or syndromes such as sarcopenia, as recently described (30). Common mechanisms of bone reabsorption are also found during aging process, i.e., an increase of the levels of pro-osteoclastogenic inflammatory cytokines such as TNF-α and IL-6, a decrease of bone-anabolic factors such as gonadal hormones and adrenal androgens as previously reviewed (193). Increased C-reactive protein was linked to an augmented fracture rate due to osteoporosis (194, 195), and circulating levels of inflammatory markers predict change in bone mineral density and reabsorption in older adults (196).

Age-related macular degeneration (AMD) is a highly prevalent, multifactorial, polygenic, and complex retinal degenerative disease. It is now widely accepted that inflammation, inflammasome activation (20), and immune system play important roles in AMD pathogenesis (197), but recently inflammaging was proposed to give a crucial contribution in the onset of AMD (198–200). Furthermore, the interplay and cross talk between protein homeostasis, autophagy, the proteasome, and heat shock proteins (HSPs) in the pathogenesis of AMD has become increasingly investigated over the past few years and has been recently reviewed (201). The role of HSPs as gatekeepers of proteolytic pathways in the retinal pigment epithelium and the implications of the disruption of the HSP-mediated chaperone functions affecting autophagy regulation, accumulation of oxidative stress-induced damage, protein aggregation and lipofuscinogenesis have also been reviewed (202) as zwell as the inflammatory process and the insufficient tissue repair (203).

### Genetic Syndromes Characterized by Accelerated Aging: A Focus on DS

One could reason that in genetic syndromes characterized by accelerated aging, the same molecular mechanisms involved in normal aging should be affected by similar, yet more precocious and intense, alterations. Actually, these syndromes, including mandibuloacral dysplasia (MADA and MADB) (204), Werner syndrome (4), and Hutchinson–Gilford progeria (2) are the subjects of intense research to understand whether the aging phenotype observed in the affected patients is superimposable to the normal one or rather it has peculiar features. In this section, we will focus on DS, which is the most common genetic cause of intellectual disability, caused by a partial or complete trisomy of chromosome 21. Life expectancy of DS persons has dramatically increased in the last two generations, and in 1988, it was calculated that about 44.4 and 13.6% of live born DS persons would survive to 60 and 68 years, respectively (205). A decade after, the average death age was 55.8 years (206). Nowadays the median life expectancy is about 60 years (207), and it is expected to further increase in the near future (208). This unprecedented increase of life expectancy, together with the early occurrence of age-related disorders let emerge a brand new phenomenon: the aging of DS persons. Actually, clinical and experimental findings lead support to the concept that DS has to be considered a premature aging syndrome, especially as far as the nervous system is concerned.

Dementia appears to be the most relevant health problem of adult DS persons, as it is the most important disorder related to mortality, together with mobility restrictions, visual impairment, and epilepsy. In addition, level of intellectual disability and institutionalization are associated with mortality (209). At the age of 50, typical neuropathological hallmarks of AD appear in DS persons, including deposition of senile plaques containing amyloid β-peptide (Aβ), neurofibrillary tangles composed of hyperphosphorylated tau-P, and cholinergic and serotoninergic reduction (210). However, signs of cognitive decline appear much earlier and are detectable already at 35–40 years of age (5, 211). This is due at least in part to the fact that APP gene is located in chromosome 21; however, other mechanisms are likely involved including endosomal–lysosomal pathway and autophagy (212). Similarly, to what occurs in the aging process, autophagy (and mitophagy in particular) is decreased in cells from DS persons, due to impaired lysosomal acidification and protease activity (212, 213).

The other major system affected by premature senescence in DS subjects is the immune system. Actually adult DS persons display a series of changes that largely recapitulate the normal aging process of the immune system. In particular, diminished NK activity (214), erosion of telomeres in T lymphocytes (215), decreased response to mitogenic stimuli of blood leukocytes (216), increased risk of autoimmune disorders (217), and decreased number of T and B lymphocytes (218). However, these commonalities with normal immunosenescence have also been interpreted as an intrinsic immunodeficiency typical of DS rather than a precocious senescence of the immune system (218). Another striking commonality with normal immunosenescence is the pro-inflammatory profile of cytokine production observed in PBMC from DS, including the increased production of IFN-γ, TNF-α, and IL-2 (219) and the increased plasmatic levels of IL-6, IL-10, TNF-α, and metalloproteases (220). This strongly resembles the phenomenon of inflammaging in old persons (39).

Down syndrome displays other typical age-associated alterations such as increased oxidative burden due to mitochondrial dysfunction (221), and, recently, it has been demonstrated that this defect can be partially restored by a treatment with metformin, a drug able to reactivate mitochondrial biogenesis by acting on the transcriptional coactivator PGC-1a (222).

As a whole, these data suggest that DS is a segmental syndrome where at least two main systems devoted to body homeostasis, i.e., the nervous and the immune systems, are affected by a premature decline that largely recapitulates what occurs in normal aging. Further support to this idea came from studies on markers of biological age (see next paragraph). In particular, analyses conducted with two types of biomarkers reliably correlated with biological age, i.e., DNA methylation age and GlycoAgeTest (see below) showed that 1. Tissues from DS persons are characterized by levels of DNA methylation typical of persons that are on average 7 years older (223); 2. The age-sensitive N-glycan species identified as GlycoAgeTest displayed accelerated dynamics in DS persons vs non-trisomic, age-matched sibs (224).

## Markers of Biological Age

Within this frame, there is growing interest around biomarkers of biological age. Biological age is intended as a synthetic index constituted by a single marker or the combination of few biological markers which, alone or integrated with functional markers, not only correlates with chronological age but is/are capable of identifying individuals “younger” or “older” than their chronological age in the same demographic cohorts.

With such biomarkers, it should be possible to obtain trajectories of aging, where the “accelerated” ones would predict unhealthy aging and diseases, while the “decelerated” ones would be associated with healthy aging and longevity. The possibility to draw trajectories of aging is a fascinating, far-reaching perspective, especially in consideration of the abovementioned long incubation preclinical period that characterizes most of the major age-related chronic diseases, and is considered the critical time window for effective treatments. Biomarkers of biological age could greatly contribute to identify the subjects characterized by higher risk to develop overt clinical diseases who would have a major benefit from tailored preventive treatments. However, these biomarkers are apparently informative about the status of deep molecular mechanisms (the seven pillars) underpinning the age-related decline which predisposes to ARDs but do not tell us which specific disease people characterized by accelerated biological age are predisposed to. Accordingly, a major biomedical aim is to identify the subjects at higher risk for each *specific* ARD at very early stage. At present, the combination of the new generation of effective biomarkers, capable of assessing the deep biological age, with the classical and innovative biochemical and functional disease-specific ones represents the best strategy to identify disease-specific aging trajectories. Within this perspective, particular attention has to be devoted to the genetics of each individual which is the complex result of the interaction between nuclear and mitochondrial genetics (stable with the exception of somatic mutations) and microbiomes’s genetics (malleable and adaptative to the environment), focusing on GM for its capability to be modified by basic habits such as nutrition. In particular, we predict that it will be useful to combine the abovementioned integrated biomarkers’ assessment with established and new genetic risk factors for ARDs, taking into account some criticalities related to population genetics and demographic birth cohorts (225).

To date, there are no clinically validated markers of biological age; however, a number of promising candidates have been proposed in the last years. We will discuss three of them: (i) DNA methylation markers, (ii) N-glycan markers, and (iii) GM biomarkers.

### DNA Methylation Markers

DNA methylation variability gained a central position in the rush for the setup of markers of biological age since several years. In a seminal paper of 2005, Fraga et al. (226) showed for the first time that in human the DNA methylation patterns change profoundly with age. With the advent of microarray technology capable to quantify the DNA methylation levels in hundreds thousands of CpG sites across the genome, the knowledge regarding variability and dynamics of such molecules increased dramatically. In particular, DNA methylation proved to be a powerful source of robust biomarkers capable to correlate with different clinical conditions (227, 228). One of the most striking results from these epigenetic studies on human models is the occurrence of directional (229–231) and stochastic (232) DNA methylation changes that highly correlate with chronological age. These observations paved the way to the generation of a number of “methylation clocks” that result from the combination of different CpG sites whose methylation level correlates with chronological age. Many of such clocks have been developed for forensic applications (233–235), thus highlighting the elevated accuracy of the chronological age estimation that can be obtained from DNA methylation data.

Of all the different clocks, three have been tested thoroughly as possible markers of biological age: the one developed by Horvath (230), the one by Hannum et al. (231), and the one by Weidner et al. (236). To date, Horvath’s DNA methylation clock provided the most interesting results as marker of biological age. This is probably due to the fact that is the only one that is applicable to all the tissues, whereas the other two clocks are validated only in blood. In many different studies, Horvath’s clock has proven to grasp features of accelerated aging in many different age-related clinical conditions such as cancer (237, 238), neurodegeneration (239–241), progeroid genetic syndromes other than DS, such as Werner syndrome (242), and all-cause mortality (243, 244). Moreover, this clock was able to show a signature of decelerated aging in human models of longevity, such as Italian centenarians and their offspring (245, 246).

Despite such promising results, a lot of work has yet to be done to include such evaluation of the biological age in the clinical practice. In this perspective, it is necessary to devote a great effort in the definition of epigenetic markers of biological age that rely on the analysis of a limited number of CpG sites to obtain an inexpensive clock suitable for large scale screenings. Indeed, both Horvath’s and Hannum’s clocks are based on the analysis of many CpG sites (353 and 75, respectively), with elevated costs that prevent their use in large scale for broad applications.

### Glycomic Biomarkers

The relative quantification of the N-glycan species that constitute the sugar shell of circulating proteins is a wealthy source of reliable biomarkers. The characterization of circulating N-glycans from sera or plasma, hereafter referred as glycomics, has provided markers in several clinical fields such as hepatology (247–249), type 2 diabetes (250–254), RA (255–258), and cancer (259–262).

It is noteworthy to mention that in a 2011 study, Vanhooren et al. showed that the glycomic parameters are correlated with age also in mice (263). In particular, studying a short-living mice model, i.e., mice defective in klotho gene expression (kl/kl), a long-living one, i.e., slow-aging Snell Dwarf mice (dw/dw) and ice fed at calorically restricted diet they showed that the N-glycan variance catch the accelerated aging of the short-living mice and the decelerated aging of the long-living ones, demonstrating that the N-glycan profiling is a promising markers of biological age also for the mice model, thus representing a powerful tool to bridge preclinical and clinical studies on aging. In the same study, the author showed that the mechanism at the basis of the age-related N-glycan changes is likely due to the impairment of the liver glycosylation machinery.

A study by Borelli et al. (224) provided the characterization of the glycomic profile of DS persons (DS). In the study the, author obtained the glycomic quantification of DS by means of DSA-FACE protocol and of the high-throughput protocol of matrix-assisted laser desorption ionization-time-of-flight-mass spectrometry. With the combination of these two protocols, the authors were able to provide for the first time the specific glycomic signature of DS and showed that the age-sensitive N-glycan species show accelerated dynamics in DS vs non-trisomic siblings and mothers.

In a study on a Netherland model of familial longevity (264, 265), the authors reported that the glycomic profile showed features of decelerated biological age, correlated with metabolic health and cardiovascular events.

Finally, it has been suggested that the age-related glycomic changes could be a contributor to inflammaging by affecting IgG structure and function. In fact, IgGs devoid of terminal galactose residue in the di-antennary N-glycan at asparagine 297 (also called IgG-G0) can exert pro-inflammatory effects through a more efficient activation of complement’s lectin pathway and phagocytosis, and their production is increased with age (266).

### Gut Microbiota Biomarkers

The comparison of GM among young adults, elderly persons, and centenarians has highlighted that the mutualistic changes in the composition and diversity of the gut ecosystem do not follow a linear relation with age, remaining highly similar from young adults to 70-year-old persons while markedly changing in centenarians. Thus, GM seems to rest in a stable state from the 3rd to the 8th decade of life (174), while after 100 years of symbiotic association with the human host, it shows a profound, and possibly adaptive, remodeling. Centenarians stand out as a separate population, as their GM shows high diversity in terms of species composition (173). In centenarians, there is a shrinkage of dominant symbiotic bacterial taxa that is counterbalanced by an increase in longevity-adapted and possibly health-promoting subdominant species (e.g., *Akkermansia, Bifidobacterium*, and *Christensenellaceae*) (267). On the other hand, GM dysbiosis has been associated with several diseases suggesting that alteration of its composition may be involved in disease-related mechanisms (268).

A recent paper addressed the potential interaction between biological age and GM. The authors identified both global and specific changes in the GM that were closely associated with biological age but not chronological age (269), suggesting that GM could be used as a potential biomarker of age.

Overall, epigenetic (DNA methylation), glycomic, and GM markers seem to be valuable markers of biological age and promising tools to draw informative aging trajectories. Many other molecular parameters obtained in particular from –omic analyses are at present under evaluation for their possible use as markers of biological age. To this regard, it is worth mentioning studies on metabolomics (32), lipidomics (270), circulating nucleic acids, in particular miRNA (271) and cell-free mtDNA (272), and metagenomics (176) that showed complex age-related reshapes in both healthy elderly and ARDs.

## Conclusion

The complex scenario emerging from the previous sections deserves and stimulates two different, even if complementary, types of conclusions. The former refers to the biomedical and molecular aspects, while the latter faces the philosophical, societal, and ethical implications and problems rose by the conceptualization here presented.

### Biomedicine and Biology

A debate exists on whether aging is a disease in itself. Some authors suggest that physiological aging (or senescence) is not really distinguishable from pathology (273), while others argue that aging is different from age-related diseases and other pathologies (274, 275). It is interesting to stress that the answer to this question has important theoretical and practical consequences, taking into account that various strategies capable of setting back the aging clock are emerging (276–278). The most relevant consequence is that, if we agree that aging is equal to disease, all human beings have to be considered as patients to be treated, being an open question when this treatment should start. As we tried to summarize in this review, many mechanisms proposed to cause aging are the same as those known to underlie ARDs/GSs, lending support to the hypothesis that the aging phenotype and ARDs/GSs are not separate entities but rather the visible consequences of the same processes which likely proceed at different rates.

Within this conceptual framework, it can be somehow puzzling to pigeonhole the phenomenon of longevity, which is a peculiar manifestation of aging. Longevity can indeed be considered the consequence of successful aging. So, why the same molecular mechanisms should lead to successful aging and longevity on one side and to unsuccessful aging and ARDs on the other? To further complicate the picture there is another important aspect, not discussed in this review, that should be, however, taken into account, i.e., the influence of gender on aging, longevity and ARDs. It is known that females have a survival advantage in advanced age, paradoxically characterized by a worse quality of life (279). In fact, females have an increased prevalence of many ARDs, in particular degenerative diseases and consequently an augment of disability. Therefore, men and women follow different trajectories to reach extreme longevity, have a diverse quantitative chance to attain longevity and the aging process is likely qualitatively different between genders (280). Several studies have also shown that sex hormones play a role in the host–microbiota interaction. Indeed, the term “microgenderome” defines the potential mediating and modulatory role of sex hormones on GM function and composition with implication for autoimmune and neuroimmune conditions (281).

**Figure 1:**
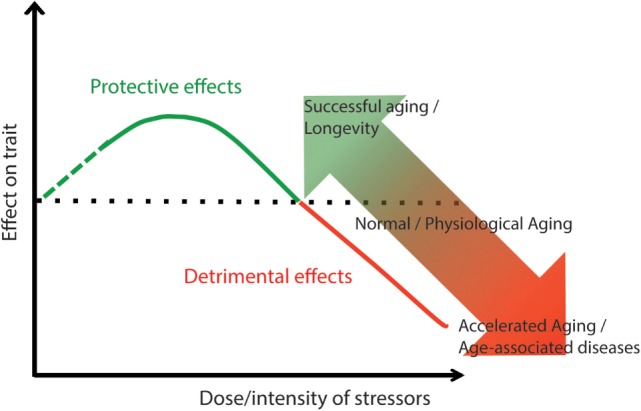
Hormesis as a possible mechanism to account for the continuum between healthy aging and geriatric syndromes (GSs)/ARDs. Lifelong low-intensity stressors stimulate maintenance and repair mechanisms with positive effects for health. The increase of stressors’ intensity can overcome the capability of the organs and systems to adapt and end up with detrimental effects (GSs/ARDs).

**Figure 2:**
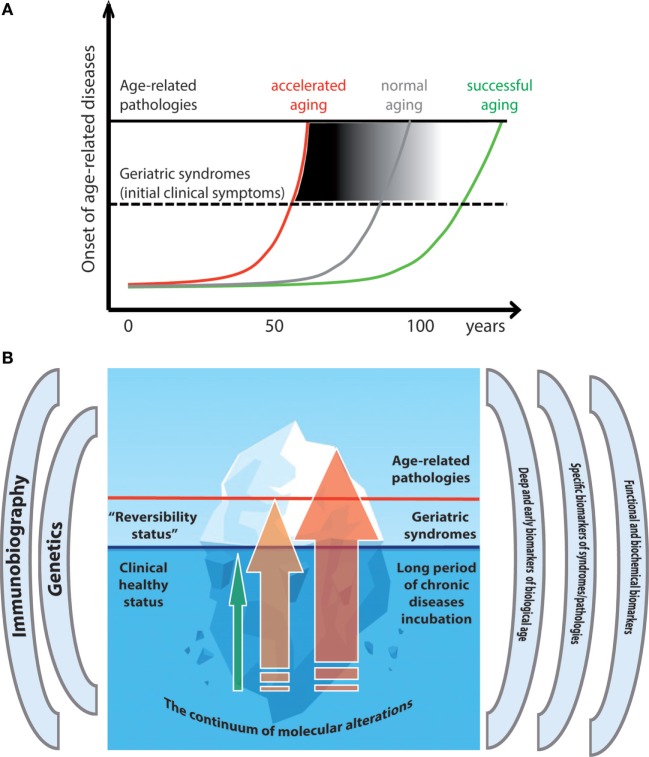
Trajectories of healthy and unhealthy aging. **(A)** The different age-trajectories are depicted as lines with different slopes, each corresponding to a rate of response and adaptation to lifelong stressors, leading to accelerated, normal, or successful aging, and reaching the threshold for ARDs at different age. **(B)** The metaphor of the iceberg is used to illustrate the continuum between healthy aging and geriatric syndromes (GSs)/ARDs. The hidden part of the iceberg illustrates the long incubation period during which no clinical signs are apparent but markers of biological age can be accelerated. Green arrow: persons with slow-aging trajectory who do not develop (or pospone) GSs/ARSs; Orange arrow: persons with faster aging who develop GSs; Red arrow: persons with accelerated aging who develop ARDs.

The overall conceptual framework of the relationship between aging and ARDs/GSs, here presented, fits quite well into the concept of hormesis, which is considered an overarching conceptualization of aging and longevity (159, 282, 283). It is known that a stressful stimulus can determine both detrimental and positive effects depending on its intensity. If the intensity of the stress is low, the response of biological system (cell, organ, or whole organism) can produce benefits that overcome the damage caused by the stress (283–285). It is possible to apply this paradigm also to the aging process (Figure 1). If the intensity of the stresses (oxidative stress, inflammation, proteostatic stress, telomere attrition, etc.) does not exceed the threshold after which the detrimental effects of such stress are higher than the adaptive, protective effects of the organismal stress response, it is likely that a successful aging will follow. A corollary of this hypothesis is that low stress is better than no stress at all, as absence of stress likely does not trigger protective effects (286). Another corollary is that the more effective is the response to stress, the higher is the level of stress intensity that can be tolerated. If an individual succeeds in maintaining his/her responses as much as possible within the range of the “hormetic zone” (green line of Figure 1), his/her trajectory toward clinical symptoms and overt disease(s) will be delayed (Figure 2A, green line). On the contrary, strong detrimental effects will accelerate aging as well as the onset of chronic diseases (red line of Figure 2A). We recently argued that the adaptive hormetic paradigm could be applied to inflammaging (287) as well as to lifestyle such as Mediterranean diet, which counteract the deleterious effects of inflammaging (283).

Which are the determinants that make the aging trajectories depicted in Figure 2 more or less steep? First of all, the environmental conditions (intensity and types of stressors, as mentioned), but also and likely most important, the capacity of the body to respond and adapt to these stressors. This capacity is determined at least in part by the individual genetic background and by epigenetic changes mediating many phenomena of adaptation and remodeling. In any case, the processes underpinning the aging progression and the corresponding successful or unsuccessful adaptive mechanisms take time, and the eventual onset of clinically overt ARDs/GSs has a long period of incubation, preceded by years/decades of deep/hidden molecular and cellular alterations, which are difficult to pinpoint with present technology and knowledge. This situation is represented in the cartoon of Figure 2B, where the continuum among healthy status, GSs, and ARDs is represented as an iceberg. The tip of the iceberg is just the (clinically) visible part of a much longer process that goes from normality to pathology. Few persons like centenarians manage to remain “healthy,” in the sense that they avoided or largely postponed the onset of ARDs/GSs even at old or very old age (green arrow), others proceed to GSs (orange arrow), while the majority develop ARDs (red arrow). Within this perspective, even “healthy” centenarians do not escape the physiological decline, and the accumulation of molecular scars that accompanies aging, but the rate of such processes is slow enough to let them stay below the threshold over which clinically overt pathologies ensue. We predict that biomarkers based on CpG DNA methylation as well as N-glycan profiling and GM composition are currently the most appropriate and powerful to distinguish biological vs chronological age and to measure the deep alterations that anticipate clinical symptoms. However, further studies are needed to assess the aging rate at the level of the various organs and systems of the body, in the same individual as required by personalized and precision medicine. Finally, beside the molecular mechanisms shared between aging and ARDs/GSs discussed earlier, a deeper level involving even more basic mechanisms (entropy failure) is likely present and will be the topic of future investigations.

### Philosophical, Ethical, and Social Implications

The second conclusion is that medicine should combat aging to combat many ARDs at a time and not one by one. In this perspective, one could envisage following two possible strategies to attain this result:

(A) Try to slow the aging rate through changes in life style, and possibly drugs or medical treatments that counteract the impairment of the abovementioned mechanisms (the seven pillars and maybe others). This strategy should help people to stay healthy and active as long as possible and pospone ARDs for decades, ideally until the apparently inevitable limit of human lifespan (288).
(B) More radically, try to rejuvenate human tissues, organs, and whole body. In this case, also the abovementioned limits of human lifespan could be likely overtaken.

We are relatively ready to the first strategy that appears more feasible and acceptable from an ethical and social point of view, as it would be very similar to what is already happening nowadays, i.e., an increase in life expectancy and in the number of people who attain 90 or 100 years of age and more in good health. Even a very long life for most people will engender various biomedical and societal problems, but this strategy has the advantage of being doable and allowing people to live longer and healthier, relieving burden from families and welfare states and, most of all, avoiding suffering, disability, and dependence.

We are instead not yet ready, in particular from a social and ethical point of view, for the second strategy, which opens uncanny scenarios of rejuvenating bodies and very long life for the bulk of the population, a topic addressed in utopian, dystopian, and science fiction novels. Taking into account, the fantastic, unprecedented rate of scientific discoveries in the field of aging and rejuvenation, it is timely and urgent to open a large debate, involving first of all the general public but also experts in different fields (economy, demography, philosophy, religion bioethics, among others). Indeed, such sensitive topics as doable age-prolongation and rejuvenation have been either neglected or conceptualized according to the scanty scientific knowledge available until recently, i.e., incomparably less than that available today and likely in the next future.

## Author Contributions

CF conceived, designed, and coordinated the writing of the whole manuscript; PG, CM, MaC, AS, AG, DM, MiC, and SS revised literature and wrote the different parts of the manuscript; all the authors contributed to critically revise and approve the final version of this manuscript.

## Conflict of Interest Statement

The authors declare that the research was conducted in the absence of any commercial or financial relationships that could be construed as a potential conflict of interest.

## Appendix

### The History of Old Age As a Disease

The first theories of aging that appeared in ancient Greece and recovered during the Middle Age identified old age as a consequence of the gradual consumption of the innate heat with the inevitable loss of body moisture, according to Hippocrates’ system of four humors (V BC). For Hippocrates (c. 460–c. 370 BC) every organism is born with a certain quantity of innate latent warmth (*calor innatus*), which progressively declines conducting to natural death (289).

Influenced by this theory, Aristotle’s (384–322 BC) on youth and old age sees old age itself as a *morbus* (disease) or a *marasmus*. To represent such an ineluctable process of degeneration, Aristotle used the image of a lamp in which the life fuel has run out, a metaphor that enjoyed a wide currency in medical literature over the centuries (290, 291).

Combining Hippocratic medicine with Aristotelian theory, Galen’s (AD 129–c. AD 199) *De Sanitate Tuenda*—where the term “gerocomy” first appeared—avoided to consider old age as a disease and rather described the health of elders as incomplete and correspondent to convalescents. More specifically, Galen saw senescence as a heterogeneous and postponable process since he observed that aging was an event impacting on population differently according to individual past history, lifestyle, and illnesses, and that by respecting a certain dietary regimen the arrival of aging might be retarded (292).

The old-age-as-a-disease idea was also present in ancient Rome. It can be detected in the comedy *Phormio* (161 BC) by the playwright Terence where the old Chremes affirms about his suffering that “the illness is old age itself,” in Seneca (c. 4 BC–AD 65) who referred to old age as an incurable illness, and in Cicero’s (106 BC–7 December 43 BC) *De Senectute* where the author argues that “we have to fight against aging, as we do against a disease” (291).

The idea that senescence was itself an illness and the image of the aged body as a consuming lamp was two of the main themes around which research into aging revolved from classical to medieval speculations, from Renaissance to eighteenth century.

Like Galen, the great Arab physician Avicenna (980–1037) refused to consider aging and death like a pathological entities, for he looked them as a result of a natural decrease of the *calor innatus* due to the consumption of the *humidum radicale*. Indeed, he was skeptical about the possibilities of medicine of retarding the aging process and then considered prolongevity not an appropriate medical goal (293). In the last centuries of the Middle Ages, of the two main medical schools of Salerno and Montpellier, the latter concentrated on the importance of the equilibrium between the four humors and the innate heat to enjoy a delayed and unimpaired senescence (294).

Interestingly, the unknown author of De retardatione accidentium senectutis (often ascribed to Roger Bacon, 1219–1292) in the thirteenth century realized that aging process can be identified with some characteristics (i.e., gray hairs), and consequently suggested that if such phenomena (*accidentia senectutis*) were to occur in adolescence, they would be called illnesses (291).

By around 1500, the old-age-illness became a prominent literary cliché that can be found both in Erasmus of Rotterdam’s poem for the Basel physician Guilielmus Copus and in Martin Luther’s comments of Ecclesiastes where he declares: “old age is *per se* a disease” (291).

During the eighteenth century, the most influential concept for the medicalization of old age was that of “*marasmus*,” a concept related to wasting fever or exhaustion, which can be traced back to Galen (295). Referring to the deterioration of old people, the marasmus senilis was a generalized pathological state that was not the effect of a temporary illness and might occur without fever. Yet, this prevailing theoretical context did not impede to realize, as well illustrated by the German physician Burkhard Seiler (1779–1843) in his seminal work *Anatomia corporis humani senilis specimen* (1799), that most old people did not die because of the weaknesses of age or senility, but rather as a result of several illnesses with a cumulative mutual effect (291), a concept that will reappear in many major theories of aging in the twentieth century (289).

## References

[1] Martin GM Genetic syndromes in man with potential relevance to the pathobiology of aging. Birth Defects Orig Artic Ser (1978) 14(1):5–39. 147113

[2] Lattanzi G Ortolani M Columbaro M Prencipe S Mattioli E Lanzarini C Lamins are rapamycin targets that impact human longevity: a study in centenarians. J Cell Sci (2014) 127(Pt 1):147–57. 10.1242/jcs.133983 24155329

[3] Cenni V D’Apice MR Garagnani P Columbaro M Novelli G Franceschi C Mandibuloacral dysplasia: a premature ageing disease with aspects of physiological ageing. Ageing Res Rev (2017) 42:1–13. 10.1016/j.arr.2017.12.001 29208544

[4] Guastafierro T Bacalini MG Marcoccia A Gentilini D Pisoni S Di Blasio AM Genome-wide DNA methylation analysis in blood cells from patients with Werner syndrome. Clin Epigenetics (2017) 9:92. 10.1186/s13148-017-0389-4 28861129 PMC5577832

[5] Ghezzo A Salvioli S Solimando MC Palmieri A Chiostergi C Scurti M Age-related changes of adaptive and neuropsychological features in persons with Down Syndrome. PLoS One (2014) 9(11):e113111. 10.1371/journal.pone.0113111 25419980 PMC4242614

[6] Kennedy BK Berger SL Brunet A Campisi J Cuervo AM Epel ES Geroscience: linking aging to chronic disease. Cell (2014) 159(4):709–13. 10.1016/j.cell.2014.10.039 25417146 PMC4852871

[7] Swardfager W Lanctôt K Rothenburg L Wong A Cappell J Herrmann N. A meta-analysis of cytokines in Alzheimer’s disease. Biol Psychiatry (2010) 68(10):930–41. 10.1016/j.biopsych.2010.06.012 20692646

[8] Nunomura A Perry G Aliev G Hirai K Takeda A Balraj EK Oxidative damage is the earliest event in Alzheimer disease. J Neuropathol Exp Neurol (2001) 60(8):759–67. 10.1093/jnen/60.8.759 11487050

[9] Moslemnezhad A Mahjoub S Moghadasi M Altered plasma marker of oxidative DNA damage and total antioxidant capacity in patients with Alzheimer’s disease. Casp J Intern Med (2016) 7:88–92. PMC4913710 27386059

[10] Mishto M Bellavista E Santoro A Stolzing A Ligorio C Nacmias B Immunoproteasome and LMP2 polymorphism in aged and Alzheimer’s disease brains. Neurobiol Aging (2006) 27(1):54–66. 10.1016/j.neurobiolaging.2004.12.004 16298241

[11] Fielder E von Zglinicki T Jurk D. The DNA damage response in neurons: die by apoptosis or survive in a senescence-like state? J Alzheimers Dis (2017) 60(s1):S107–31. 10.3233/JAD-161221 28436392

[12] Bekkering P Jafri I van Overveld FJ Rijkers GT. The intricate association between gut microbiota and development of type 1, type 2 and type 3 diabetes. Expert Rev Clin Immunol (2013) 9(11):1031–41. 10.1586/1744666X.2013.848793 24138599

[13] Krtolica A Parrinello S Lockett S Desprez PY Campisi J. Senescent fibroblasts promote epithelial cell growth and tumorigenesis: a link between cancer and aging. Proc Natl Acad Sci U S A (2001) 98:12072–7. 10.1073/pnas.211053698 11593017 PMC59769

[14] Liu D Hornsby PJ. Senescent human fibroblasts increase the early growth of xenograft tumors via matrix metalloproteinase secretion. Cancer Res (2007) 67:3117–26. 10.1158/0008-5472.CAN-06-3452 17409418

[15] Sansone P Storci G Tavolari S Guarnieri T Giovannini C Taffurelli M IL-6 triggers malignant features in mammospheres from human ductal breast carcinoma and normal mammary gland. J Clin Invest (2007) 117(12):3988–4002. 10.1172/JCI32533 18060036 PMC2096439

[16] Hanahan D Weinberg RA Hallmarks of cancer: the next generation. Cell (2011) 144(5):646–74. 10.1016/j.cell.2011.02.013 21376230

[17] MacNee W. Is chronic obstructive pulmonary disease an accelerated aging disease? Ann Am Thorac Soc (2016) 13(Suppl_5):S429–37. 10.1513/AnnalsATS.201602-124AW 28005421

[18] Barnes PJ. Senescence in COPD and its comorbidities. Annu Rev Physiol (2017) 79:517–39. 10.1146/annurev-physiol-022516-034314 27959617

[19] Iannaccone A Giorgianni F New DD Hollingsworth TJ Umfress A Alhatem AH Circulating autoantibodies in age-related macular degeneration recognize human macular tissue antigens implicated in autophagy, immunomodulation, and protection from oxidative stress and apoptosis. PLoS One (2015) 10(12):e0145323 10.1371/journal.pone.0145323 26717306 PMC4696815

[20] Kerur N Fukuda S Banerjee D Kim Y Fu D Apicella I cGAS drives noncanonical-inflammasome activation in age-related macular degeneration. Nat Med (2018) 24(1):50–61. 10.1038/nm.4450 29176737 PMC5760363

[21] Loeser RF Collins JA Diekman BO Aging and the pathogenesis of osteoarthritis. Nat Rev Rheumatol (2016) 12(7):412–20. 10.1038/nrrheum.2016.65 27192932 PMC4938009

[22] Straub RH Cutolo M Pacifici R Evolutionary medicine and bone loss in chronic inflammatory diseases – a theory of inflammation-related osteopenia. Semin Arthritis Rheum (2015) 45(2):220–8. 10.1016/j.semarthrit.2015.04.014 26044543 PMC4570856

[23] Chinta SJ Lieu CA Demaria M Laberge RM Campisi J Andersen JK Environmental stress, aging and glial cell senescence: a novel mechanistic link to Parkinson’s disease? J Intern Med (2013) 273(5):429–36. 10.1111/joim.12029 23600398 PMC3633085

[24] Tremlett H Bauer KC Appel-Cresswell S Finlay BB Waubant E. The gut microbiome in human neurological disease: a review. Ann Neurol (2017) 81(3):369–82. 10.1002/ana.24901 28220542

[25] Rogers GB Keating DJ Young RL Wong ML Licinio J Wesselingh S. From gut dysbiosis to altered brain function and mental illness: mechanisms and pathways. Mol Psychiatry (2016) 21(6):738–48. 10.1038/mp.2016.50 27090305 PMC4879184

[26] Potempa J Mydel P Koziel J. The case for periodontitis in the pathogenesis of rheumatoid arthritis. Nat Rev Rheumatol (2017) 13(10):606–20. 10.1038/nrrheum.2017.132 28835673

[27] Taniguchi N Kawakami Y Maruyama I Lotz M HMGB proteins and arthritis. Hum Cell (2017) 31(1):1–9. 10.1007/s13577-017-0182-x 28916968 PMC6541443

[28] van der Poel C Gosselin LE Schertzer JD Ryall JG Swiderski K Wondemaghen M Aging prolongs inflammatory marker expression in regenerating rat skeletal muscles after injury. J Inflamm (Lond) (2011) 8(1):41 10.1186/1476-9255-8-41 22206492 PMC3339359

[29] Costamagna D Costelli P Sampaolesi M Penna F. Role of inflammation in muscle homeostasis and myogenesis. Mediators Inflamm (2015) 2015:805172. 10.1155/2015/805172 26508819 PMC4609834

[30] Wilson D Jackson T Sapey E Lord JM. Frailty and sarcopenia: the potential role of an aged immune system. Aging Res Rev (2017) 36:1–10. 10.1016/j.arr.2017.01.006 28223244

[31] Franceschi C Valensin S Bonafè M Paolisso G Yashin AI Monti D The network and the remodeling theories of aging: historical background and new perspectives. Exp Gerontol (2000) 35(6–7):879–96. 10.1016/S0531-5565(00)00172-8 11053678

[32] Collino S Montoliu I Martin FP Scherer M Mari D Salvioli S Metabolic signatures of extreme longevity in northern Italian centenarians reveal a complex remodelling of lipids, amino acids, and gut microbiota metabolism. PLoS One (2013) 8(3):e56564 10.1371/journal.pone.0056564 23483888 PMC3590212

[33] Capri M Salvioli S Sevini F Valensin S Celani L Monti D The genetics of human longevity. Ann N Y Acad Sci (2006) 1067:252–63. 10.1196/annals.1354.033 16803995

[34] Tan Q Kruse TA Christensen K Design and analysis in genetic studies of human aging and longevity. Aging Res Rev (2006) 5(4):371–87. 10.1016/j.arr.2005.10.002 16337437

[35] Capri M Santoro A Garagnani P Bacalini MG Pirazzini C Olivieri F Genes of human longevity: an endless quest? Curr Vasc Pharmacol (2014) 12(5):707–17. 10.2174/1570161111666131219110301 24350927

[36] Kirkwood TB Feder M Finch CE Franceschi C Globerson A Klingenberg CP What accounts for the wide variation in life span of genetically identical organisms reared in a constant environment? Mech Aging Dev (2005) 126(3):439–43. 10.1016/j.mad.2004.09.008 15664632

[37] Blagosklonny MV Hall MN. Growth and aging: a common molecular mechanism. Aging (Albany NY) (2009) 1(4):357–62. 10.18632/aging.100040 20157523 PMC2806018

[38] Blagosklonny MV Aging is not programmed: genetic pseudo-program is a shadow of developmental growth. Cell Cycle (2013) 12(24):3736–42. 10.4161/cc.27188 24240128 PMC3905065

[39] Franceschi C Bonafè M Valensin S Olivieri F De Luca M Ottaviani E Inflamm-aging. An evolutionary perspective on immunosenescence. Ann N Y Acad Sci (2000) 908:244–54. 10.1111/j.1749-6632.2000.tb06651.x 10911963

[40] Franceschi C Garagnani P Vitale G Capri M Salvioli S Inflammaging and ‘Garb-aging’. Trends Endocrinol Metab (2017) 28(3):199–212. 10.1016/j.tem.2016.09.005 27789101

[41] Coppé JP Desprez PY Krtolica A Campisi J. The senescence-associated secretory phenotype: the dark side of tumor suppression. Annu Rev Pathol (2010) 5:99–118. 10.1146/annurev-pathol-121808-102144 20078217 PMC4166495

[42] Bergman H Ferrucci L Guralnik J Hogan DB Hummel S Karunananthan S Frailty: an emerging research and clinical paradigm – issues and controversies. J Gerontol A Biol Sci Med Sci (2007) 62(7):731–7. 10.1093/gerona/62.7.731 17634320 PMC2645660

[43] Fried LP Tangen CM Walston J Newman AB Hirsch C Gottdiener J Frailty in older adults: evidence for a phenotype. J Gerontol A Biol Sci Med Sci (2001) 56(3):M146–56. 10.1093/gerona/56.3.M146 11253156

[44] Mitnitski AB Mogilner AJ Rockwood K. Accumulation of deficits as a proxy measure of aging. ScientificWorldJournal (2001) 1:323–36. 10.1100/tsw.2001.58 12806071 PMC6084020

[45] Searle SD Mitnitski A Gahbauer EA Gill TM Rockwood K. A standard procedure for creating a frailty index. BMC Geriatr (2008) 8:24. 10.1186/1471-2318-8-24 18826625 PMC2573877

[46] Aguayo GA Donneau AF Vaillant MT Schritz A Franco OH Stranges S Agreement between 35 published frailty scores in the general population. Am J Epidemiol (2017) 186(4):420–34. 10.1093/aje/kwx061 28633404 PMC5860330

[47] Theou O Cann L Blodgett J Wallace LM Brothers TD Rockwood K Modifications to the frailty phenotype criteria: systematic review of the current literature and investigation of 262 frailty phenotypes in the survey of health, aging, and retirement in Europe. Aging Res Rev (2015) 21:78–94. 10.1016/j.arr.2015.04.001 25846451

[48] Jha SR Hannu MK Newton PJ Wilhelm K Hayward CS Jabbour A Reversibility of frailty after bridge-to-transplant ventricular assist device implantation or heart transplantation. Transplant Direct (2017) 3(7):e167. 10.1097/TXD.0000000000000690 28706970 PMC5498008

[49] Michel JP Cruz-Jentoft AJ Cederholm T Frailty, exercise and nutrition. Clin Geriatr Med (2015) 31(3):375–87. 10.1016/j.cger.2015.04.006 26195097

[50] Davies B García F Ara I Artalejo FR Rodriguez-Mañas L Walter S. Relationship between sarcopenia and frailty in the toledo study of healthy aging: a population based cross-sectional study. J Am Med Dir Assoc (2017). 10.1016/j.jamda.2017.09.014 29079029

[51] Wu IC Lin CC Hsiung CA. Emerging roles of frailty and inflammaging in risk assessment of age-related chronic diseases in older adults: the intersection between aging biology and personalized medicine. Biomedicine (Taipei) (2015) 5(1):1. 10.7603/s40681-015-0001-1 25722960 PMC4333299

[52] Fried LP Ferrucci L Etiological role of aging in chronic diseases: from epidemiological evidence to the new geroscience. In: Sierra F Kohanski R, editors. Advances in Geroscience. Cham: Springer International Publishing (2015). p. 37–51.

[53] Morrisette-Thomas V Cohen AA Fülöp T Riesco É Legault V Li Q Inflamm-aging does not simply reflect increases in pro-inflammatory markers. Mech Aging Dev (2014) 139:49–57. 10.1016/j.mad.2014.06.005 25011077 PMC5881904

[54] Rutenberg AD Mitnitski AB Farrell SG Rockwood K. Unifying aging and frailty through complex dynamical networks. Exp Gerontol (2017). 10.1016/j.exger.2017.08.027 28847723

[55] Cruz-Jentoft AJ Baeyens JP Bauer JM Boirie Y Cederholm T Landi F Sarcopenia: European consensus on definition and diagnosis: report of the European working group on sarcopenia in older people. Age Aging (2010) 39:412–23. 10.1093/aging/afq034 PMC2886201 20392703

[56] Filippin LI Teixeira VN da Silva MP Miraglia F da Silva FS. Sarcopenia: a predictor of mortality and the need for early diagnosis and intervention. Aging Clin Exp Res (2015) 27:249–54. 10.1007/s40520-014-0281-4 25365952

[57] Budui SL Rossi AP Zamboni M. The pathogenetic bases of sarcopenia. Clin Cases Miner Bone Metab (2015) 12:22–6. 10.11138/ccmbm/2015.12.1.022 26136791 PMC4469221

[58] Scicchitano BM Pelosi L Sica G Musarò A. The physiopathologic role of oxidative stress in skeletal muscle. Mech Aging Dev (2017) 170:37–44. 10.1016/j.mad.2017.08.009 28851603

[59] Conboy IM Conboy MJ Wagers AJ Girma ER Weissman IL Rando TA. Rejuvenation of aged progenitor cells by exposure to a young systemic environment. Nature (2005) 433:760–4. 10.1038/nature03260 15716955

[60] Barberi L Scicchitano BM De Rossi M Bigot A Duguez S Wielgosik A Age-dependent alteration in muscle regeneration: the critical role of tissue niche. Biogerontology (2013) 14(3):273–92. 10.1007/s10522-013-9429-4 23666344 PMC3719007

[61] Baylis D Bartlett DB Patel HP Roberts HC. Understanding how we age: insights into inflammaging. Longev Healthspan (2013) 2:8. 10.1186/2046-2395-2-8 24472098 PMC3922951

[62] Tidball JG. Inflammatory processes in muscle injury and repair. Am J Physiol Regul Integr Comp Physiol (2005) 288:R345–53. 10.1152/ajpregu.00454.2004 15637171

[63] Mercken EM Capri M Carboneau BA Conte M Heidler J Santoro A Conserved and species-specific molecular denominators in mammalian skeletal muscle aging. NPJ Aging Mech Dis (2017) 3:8. 10.1038/s41514-017-0009-8 28649426 PMC5460213

[64] Beyer I Mets T Bautmans I. Chronic low-grade inflammation and age-related sarcopenia. Curr Opin Clin Nutr Metab Care (2012) 15:12–22. 10.1097/MCO.0b013e32834dd297 22108098

[65] Vitale G Salvioli S Franceschi C Oxidative stress and the aging endocrine system. Nat Rev Endocrinol (2013) 9(4):228–40. 10.1038/nrendo.2013.29 23438835

[66] Ilich JZ Kelly OJ Inglis JE. Osteosarcopenic obesity syndrome: what is it and how can it be identified and diagnosed? Curr Gerontol Geriatr Res (2016) 2016:7325973. 10.1155/2016/7325973 27667996 PMC5030469

[67] Gonnelli S Caffarelli C Nuti R. Obesity and fracture risk. Clin Cases Miner Bone Metab (2014) 11:9–14. 10.11138/ccmbm/2014.11.1.009 25002873 PMC4064448

[68] Dillin A Gottschling DE Nyström T. The good and the bad of being connected: the integrons of aging. Curr Opin Cell Biol (2014) 26:107–12. 10.1016/j.ceb.2013.12.003 24529252 PMC3927154

[69] Vaz Fragoso CA Gill TM. Respiratory impairment and the aging lung: a novel paradigm for assessing pulmonary function. J Gerontol A Biol Sci Med Sci (2012) 67(3):264–75. 10.1093/gerona/glr198 22138206 PMC3297762

[70] Mannino DM Homa DM Akinbami LJ Ford ES Redd SC Chronic obstructive pulmonary disease surveillance – United States, 1971-2000. Respir Care (2002) 47(10):1184–99. 12354338

[71] Hogg JC Timens W. The pathology of chronic obstructive pulmonary disease. Annu Rev Pathol (2009) 4:435–59. 10.1146/annurev.pathol.4.110807.092145 18954287

[72] Salvi SS Barnes PJ. Chronic obstructive pulmonary disease in non-smokers. Lancet (2009) 374(9691):733–43. 10.1016/S0140-6736(09)61303-9 19716966

[73] Gordon SB Bruce NG Grigg J Hibberd PL Kurmi OP Lam KB Respiratory risks from household air pollution in low and middle income countries. Lancet Respir Med (2014) 2(10):823–60. 10.1016/S2213-2600(14)70168-7 25193349 PMC5068561

[74] Burney P Jithoo A Kato B Janson C Mannino D Nizankowska-Mogilnicka E Burden of obstructive lung disease (BOLD) study. chronic obstructive pulmonary disease mortality and prevalence: the associations with smoking and poverty – a BOLD analysis. Thorax (2014) 69(5):465–73. 10.1136/thoraxjnl-2013-204460 24353008 PMC3995258

[75] Ito K Barnes PJ. COPD as a disease of accelerated lung aging. Chest (2009) 135(1):173–80. 10.1378/chest.08-1419 19136405

[76] Mercado N Ito K Barnes PJ Accelerated aging of the lung in COPD: new concepts. Thorax (2015) 70(5):482–9. 10.1136/thoraxjnl-2014-206084 25739910

[77] Meiners S Eickelberg O Königshoff M Hallmarks of the aging lung. Eur Respir J (2015) 45(3):807–27. 10.1183/09031936.00186914 25657021

[78] Savale L Chaouat A Bastuji-Garin S Marcos E Boyer L Maitre B Shortened telomeres in circulating leukocytes of patients with chronic obstructive pulmonary disease. Am J Respir Crit Care Med (2009) 179(7):566–71. 10.1164/rccm.200809-1398OC 19179485 PMC4850213

[79] Amsellem V Gary-Bobo G Marcos E Maitre B Chaar V Validire P Telomere dysfunction causes sustained inflammation in chronic obstructive pulmonary disease. Am J Respir Crit Care Med (2011) 184(12):1358–66. 10.1164/rccm.201105-0802OC 21885626

[80] Tsuji T Aoshiba K Nagai A. Alveolar cell senescence exacerbates pulmonary inflammation in patients with chronic obstructive pulmonary disease. Respiration (2010) 80(1):59–70. 10.1159/000268287 20016134

[81] Albrecht E Sillanpää E Karrasch S Alves AC Codd V Hovatta I Telomere length in circulating leukocytes is associated with lung function and disease. Eur Respir J (2014) 43(4):983–92. 10.1183/09031936.00046213 24311771

[82] Chilosi M Carloni A Rossi A Poletti V. Premature lung aging and cellular senescence in the pathogenesis of idiopathic pulmonary fibrosis and COPD/emphysema. Transl Res (2013) 162(3):156–73. 10.1016/j.trsl.2013.06.004 23831269

[83] John-Schuster G Günter S Hager K Conlon TM Eickelberg O Yildirim AÖ. Inflammaging increases susceptibility to cigarette smoke-induced COPD. Oncotarget (2016) 7(21):30068–83. 10.18632/oncotarget.4027 26284585 PMC5058664

[84] Johnson SC Rabinovitch PS Kaeberlein M mTOR is a key modulator of aging and age-related disease. Nature (2013) 493(7432):338–45. 10.1038/nature11861 23325216 PMC3687363

[85] Eijkelenboom A Burgering BM. FOXOs: signalling integrators for homeostasis maintenance. Nat Rev Mol Cell Biol (2013) 14(2):83–97. 10.1038/nrm3507 23325358

[86] Hwang JW Rajendrasozhan S Yao H Chung S Sundar IK Huyck HL FOXO3 deficiency leads to increased susceptibility to cigarette smoke-induced inflammation, airspace enlargement, and chronic obstructive pulmonary disease. J Immunol (2011) 187(2):987–98. 10.4049/jimmunol.1001861 21690325 PMC3131437

[87] Rubinsztein DC Mariño G Kroemer G Autophagy and aging. Cell (2011) 146(5):682–95. 10.1016/j.cell.2011.07.030 21884931

[88] Calderwood SK Murshid A Prince T The shock of aging: molecular chaperones and the heat shock response in longevity and aging – a mini-review. Gerontology (2009) 55(5):550–8. 10.1159/000225957 19546513 PMC2754743

[89] Chondrogianni N Sakellari M Lefaki M Papaevgeniou N Gonos ES. Proteasome activation delays aging in vitro and in vivo. Free Radic Biol Med (2014) 71:303–20. 10.1016/j.freeradbiomed.2014.03.031 24681338

[90] Ferrington DA Husom AD Thompson LV. Altered proteasome structure, function, and oxidation in aged muscle. FASEB J (2005) 19:644–6. 10.1096/fj.04-2578fje 15677694

[91] Chondrogianni N Stratford FL Trougakos IP Friguet B Rivett AJ Gonos ES. Central role of the proteasome in senescence and survival of human fibroblasts: induction of a senescence-like phenotype upon its inhibition and resistance to stress upon its activation. J Biol Chem (2003) 278:28026–37. 10.1074/jbc.M301048200 12736271

[92] Chondrogianni N Petropoulos I Franceschi C Friguet B Gonos ES. Fibroblast cultures from healthy centenarians have an active proteasome. Exp Gerontol (2000) 35:721–8. 10.1016/S0531-5565(00)00137-6 11053662

[93] Chondrogianni N Voutetakis K Kapetanou M Delitsikou V Papaevgeniou N Sakellari M Proteasome activation: an innovative promising approach for delaying aging and retarding age-related diseases. Aging Res Rev (2015) 23(Pt A):37–55. 10.1016/j.arr.2014.12.003 25540941

[94] Meiners S Eickelberg O. What shall we do with the damaged proteins in lung disease? Ask the proteasome! Eur Respir J (2012) 40(5):1260–8. 10.1183/09031936.00208511 22441749

[95] Ryter SW Chen ZH Kim HP Choi AM. Autophagy in chronic obstructive pulmonary disease: homeostatic or pathogenic mechanism? Autophagy (2009) 5(2):235–7. 10.4161/auto.5.2.7495 19066468

[96] Chen ZH Lam HC Jin Y Kim HP Cao J Lee SJ Autophagy protein microtubule-associated protein 1 light chain-3B (LC3B) activates extrinsic apoptosis during cigarette smoke-induced emphysema. Proc Natl Acad Sci U S A (2010) 107(44):18880–5. 10.1073/pnas.1005574107 20956295 PMC2973911

[97] Monick MM Powers LS Walters K Lovan N Zhang M Gerke A Identification of an autophagy defect in smokers’ alveolar macrophages. J Immunol (2010) 185(9):5425–35. 10.4049/jimmunol.1001603 20921532 PMC3057181

[98] Dunlop EA Tee AR. mTOR and autophagy: a dynamic relationship governed by nutrients and energy. Semin Cell Dev Biol (2014) 36:121–9. 10.1016/j.semcdb.2014.08.006 25158238

[99] Donnelly LE Barnes PJ. Defective phagocytosis in airways disease. Chest (2012) 141(4):1055–62. 10.1378/chest.11-2348 22474147

[100] Sureshbabu A Bhandari V. Targeting mitochondrial dysfunction in lung diseases: emphasis on mitophagy. Front Physiol (2013) 4:384. 10.3389/fphys.2013.00384 24421769 PMC3872744

[101] Hoffmann RF Zarrintan S Brandenburg SM Kol A de Bruin HG Jafari S Prolonged cigarette smoke exposure alters mitochondrial structure and function in airway epithelial cells. Respir Res (2013) 14:97. 10.1186/1465-9921-14-97 24088173 PMC3852998

[102] Mizumura K Cloonan SM Nakahira K Bhashyam AR Cervo M Kitada T Mitophagy-dependent necroptosis contributes to the pathogenesis of COPD. J Clin Invest (2014) 124(9):3987–4003. 10.1172/JCI74985 25083992 PMC4151233

[103] Li J Dai A Hu R Zhu L Tan S. Positive correlation between PPARgamma/PGC-1alpha and gamma-GCS in lungs of rats and patients with chronic obstructive pulmonary disease. Acta Biochim Biophys Sin (Shanghai) (2010) 42(9):603–14. 10.1093/abbs/gmq071 20732852

[104] Ryan DM Vincent TL Salit J Walters MS Agosto-Perez F Shaykhiev R Smoking dysregulates the human airway basal cell transcriptome at COPD risk locus 19q13.2. PLoS One (2014) 9(2):e88051. 10.1371/journal.pone.0088051 24498427 PMC3912203

[105] DeNardo DG Andreu P Coussens LM. Interactions between lymphocytes and myeloid cells regulate pro- versus anti-tumor immunity. Cancer Metastasis Rev (2010) 29(2):309–16. 10.1007/s10555-010-9223-6 20405169 PMC2865635

[106] Grivennikov SI Greten FR Karin M Immunity, inflammation, and cancer. Cell (2010) 140(6):883–99. 10.1016/j.cell.2010.01.025 20303878 PMC2866629

[107] Qian BZ Pollard JW. Macrophage diversity enhances tumor progression and metastasis. Cell (2010) 141(1):39–51. 10.1016/j.cell.2010.03.014 20371344 PMC4994190

[108] Karnoub AE Weinberg RA Chemokine networks and breast cancer metastasis. Breast Dis (2006) 26:75–85. 17473367 10.3233/bd-2007-26107

[109] de Visser KE Eichten A Coussens LM. Paradoxical roles of the immune system during cancer development. Nat Rev Cancer (2006) 6(1):24–37. 10.1038/nrc1782 16397525

[110] Bald T Quast T Landsberg J Rogava M Glodde N Lopez-Ramos D Ultraviolet-radiation-induced inflammation promotes angiotropism and metastasis in melanoma. Nature (2014) 507(7490):109–13. 10.1038/nature13111 24572365

[111] Coppé JP Patil CK Rodier F Sun Y Munoz DP Goldstein J Senescence-associated secretory phenotypes reveal cell-nonautonomous functions of oncogenic RAS and the p53 tumor suppressor. PLoS Biol (2008) 6(12):e301. 10.1371/journal.pbio.0060301 19053174 PMC2592359

[112] Lecot P Alimirah F Desprez PY Campisi J Wiley C. Context-dependent effects of cellular senescence in cancer development. Br J Cancer (2016) 114(11):1180–4. 10.1038/bjc.2016.115 27140310 PMC4891501

[113] Bonafè M Storci G Franceschi C. Inflamm-aging of the stem cell niche: breast cancer as a paradigmatic example: breakdown of the multi-shell cytokine network fuels cancer in aged people. Bioessays (2012) 34(1):40–9. 10.1002/bies.201100104 22086861

[114] Pang WW Schrier SL Weissman IL. Age-associated changes in human hematopoietic stem cells. Semin Hematol (2017) 54(1):39–42. 10.1053/j.seminhematol.2016.10.004 28088986

[115] Koschmieder S Mughal TI Hasselbalch HC Barosi G Valent P Kiladjian JJ Myeloproliferative neoplasms and inflammation: whether to target the malignant clone or the inflammatory process or both. Leukemia (2016) 30(5):1018–24. 10.1038/leu.2016.12 26854026

[116] Kovtonyuk LV Fritsch K Feng X Manz MG Takizawa H. Inflamm-aging of hematopoiesis, hematopoietic stem cells, and the bone marrow microenvironment. Front Immunol (2016) 7:502. 10.3389/fimmu.2016.00502 27895645 PMC5107568

[117] Chang Q Bournazou E Sansone P Berishaj M Gao SP Daly L The IL-6/JAK/Stat3 feed-forward loop drives tumorigenesis and metastasis. Neoplasia (2013) 15(7):848–62. 10.1593/neo.13706 23814496 PMC3689247

[118] Coppedè F. The epidemiology of premature aging and associated comorbidities. Clin Interv Aging (2013) 8:1023–32. 10.2147/CIA.S37213 24019745 PMC3760297

[119] Shiloh Y Lederman HM Ataxia-telangiectasia (A-T): an emerging dimension of premature aging. Aging Res Rev (2017) 33:76–88. 10.1016/j.arr.2016.05.002 27181190

[120] Pavlidis N Stanta G Audisio RA. Cancer prevalence and mortality in centenarians: a systematic review. Crit Rev Oncol Hematol (2012) 83:145–52. 10.1016/j.critrevonc.2011.09.007 22024388

[121] Boyko AA Troyanova NI Kovalenko EI Sapozhnikov AM Similarity and differences in inflammation-related characteristics of the peripheral immune system of patients with Parkinson’s and Alzheimer’s diseases. Int J Mol Sci (2017) 18(12):E2633 10.3390/ijms18122633 29211044 PMC5751236

[122] Selkoe DJ Cell biology of protein misfolding: the examples of Alzheimer’s and Parkinson’s diseases. Nat Cell Biol (2004) 6:1054–61. 10.1038/ncb1104-1054 15516999

[123] Tan JM Wong ES Lim KL. Protein misfolding and aggregation in Parkinson’s disease. Antioxid Redox Signal (2009) 11(9):2119–34. 10.1089/ARS.2009.2490 19243238

[124] Ebrahimi-Fakhari D Wahlster L McLean PJ Molecular chaperones in Parkinson’s disease – present and future. J Parkinsons Dis (2011) 1(4):299–320. 22279517 PMC3264060

[125] Bloom GS. Amyloid-β and tau: the trigger and bullet in Alzheimer disease pathogenesis. JAMA Neurol (2014) 71(4):505–8. 10.1001/jamaneurol.2013.5847 24493463 PMC12908160

[126] Wakabayashi K Tanji K Odagiri S Miki Y Mori F Takahashi H. The Lewy body in Parkinson’s disease and related neurodegenerative disorders. Mol Neurobiol (2013) 47(2):495–508. 10.1007/s12035-012-8280-y 22622968

[127] Kempuraj D Thangavel R Natteru PA Selvakumar GP Saeed D Zahoor H Neuroinflammation induces neurodegeneration. J Neurol Neurosurg Spine (2016) 1(1):1003. 28127589 PMC5260818

[128] Santoro A Siviero P Minicuci N Bellavista E Mishto M Olivieri F Effects of donepezil, galantamine and rivastigmine in 938 Italian patients with Alzheimer’s disease: a prospective, observational study. CNS Drugs (2010) 24(2):163–76. 10.2165/11310960-000000000-00000 20088621

[129] Hampel H Bürger K Teipel SJ Bokde AL Zetterberg H Blennow K. Core candidate neurochemical and imaging biomarkers of Alzheimer’s disease. Alzheimers Dement (2008) 4(1):38–48. 10.1016/j.jalz.2007.08.006 18631949

[130] Nelson PT Alafuzoff I Bigio EH Bouras C Braak H Cairns NJ Correlation of Alzheimer disease neuropathologic changes with cognitive status: a review of the literature. J Neuropathol Exp Neurol (2012) 71(5):362–81. 10.1097/NEN.0b013e31825018f7 22487856 PMC3560290

[131] Herrup K. The case for rejecting the amyloid cascade hypothesis. Nat Neurosci (2015) 18(6):794–9. 10.1038/nn.4017 26007212

[132] Musiek ES Holtzman DM. Three dimensions of the amyloid hypothesis: time, space and ‘wingmen’. Nat Neurosci (2015) 18(6):800–6. 10.1038/nn.4018 26007213 PMC4445458

[133] Kempuraj D Thangavel R Selvakumar GP Zaheer S Ahmed ME Raikwar SP Brain and peripheral atypical inflammatory mediators potentiate neuroinflammation and neurodegeneration. Front Cell Neurosci (2017) 11:216. 10.3389/fncel.2017.00216 28790893 PMC5522882

[134] Perry VH. The influence of systemic inflammation on inflammation in the brain: implications for chronic neurodegenerative disease. Brain Behav Immun (2004) 18:407–13. 10.1016/j.bbi.2004.01.004 15265532

[135] Holmes C El-Okl M Williams AL Cunningham C Wilcockson D Perry VH. Systemic infection, interleukin 1beta, and cognitive decline in Alzheimer’s disease. J Neurol Neurosurg Psychiatry (2003) 74(6):788–9. 10.1136/jnnp.74.6.788 12754353 PMC1738504

[136] Goncharova LB Tarakanov AO. Molecular networks of brain and immunity. Brain Res Rev (2007) 55(1):155–66. 10.1016/j.brainresrev.2007.02.003 17408562

[137] Benedict C Scheller J Rose-John S Born J Marshall L. Enhancing influence of intranasal interleukin-6 on slow-wave activity and memory consolidation during sleep. FASEB J (2009) 23(10):3629–36. 10.1096/fj.08-122853 19546306

[138] del Rey A Balschun D Wetzel W Randolf A Besedovsky HO A cytokine network involving brain-borne IL-1β, IL-1ra, IL-18, IL-6, and TNFα operates during long-term potentiation and learning. Brain Behav Immun (2013) 33:15–23. 10.1016/j.bbi.2013.05.011 23747799

[139] Hoshino K Hasegawa K Kamiya H Morimoto Y Synapse-specific effects of IL-1β on long-term potentiation in the mouse hippocampus. Biomed Res (2017) 38(3):183–8. 10.2220/biomedres.38.183 28637953

[140] Blasko I Veerhuis R Stampfer-Kountchev M Saurwein-Teissl M Eikelenboom P Grubeck-Loebenstein B. Costimulatory effects of interferon-gamma and interleukin-1beta or tumor necrosis factor alpha on the synthesis of Abeta1-40 and Abeta1-42 by human astrocytes. Neurobiol Dis (2000) 7(6 Pt B):682–9. 10.1006/nbdi.2000.0321 11114266

[141] Sultana R Mecocci P Mangialasche F Cecchetti R Baglioni M Butterfield DA. Increased protein and lipid oxidative damage in mitochondria isolated from lymphocytes from patients with Alzheimer’s disease: insights into the role of oxidative stress in Alzheimer’s disease and initial investigations into a potential biomarker for this dementing disorder. J Alzheimers Dis (2011) 24(1):77–84. 10.3233/JAD-2011-101425 21383494

[142] Santoro A Balbi V Balducci E Pirazzini C Rosini F Tavano F Evidence for sub-haplogroup h5 of mitochondrial DNA as a risk factor for late onset Alzheimer’s disease. PLoS One (2010) 5(8):e12037 10.1371/journal.pone.0012037 20700462 PMC2917370

[143] Mecocci P Polidori MC Cherubini A Ingegni T Mattioli P Catani M Lymphocyte oxidative DNA damage and plasma antioxidants in Alzheimer disease. Arch Neurol (2002) 59(5):794–8. 10.1001/archneur.59.5.794 12020262

[144] Sliwinska A Kwiatkowski D Czarny P Toma M Wigner P Drzewoski J The levels of 7,8-dihydrodeoxyguanosine (8-oxoG) and 8-oxoguanine DNA glycosylase 1 (OGG1) – a potential diagnostic biomarkers of Alzheimer’s disease. J Neurol Sci (2016) 368:155–9. 10.1016/j.jns.2016.07.008 27538622

[145] Schippling S Kontush A Arlt S Buhmann C Stürenburg HJ Mann U Increased lipoprotein oxidation in Alzheimer’s disease. Free Radic Biol Med (2000) 28(3):351–60. 10.1016/S0891-5849(99)00247-6 10699746

[146] Shad KF Aghazadeh Y Ahmad S Kress B. Peripheral markers of Alzheimer’s disease: surveillance of white blood cells. Synapse (2013) 67(8):541–3. 10.1002/syn.21651 23404438

[147] Benarroch EE. Neuron-astrocyte interactions: partnership for normal function and disease in the central nervous system. Mayo Clin Proc (2005) 80:1326–38. 10.4065/80.10.1326 16212146

[148] Magistretti PJ. Neuron-glia metabolic coupling and plasticity. J Exp Biol (2006) 209:2304–11. 10.1242/jeb.02208 16731806

[149] Bhat R Crowe EP Bitto A Moh M Katsetos CD Garcia FU Astrocyte senescence as a component of Alzheimer’s disease. PLoS One (2012) 7(9):e45069. 10.1371/journal.pone.0045069 22984612 PMC3440417

[150] Caracciolo B Xu W Collins S Fratiglioni L. Cognitive decline, dietary factors and gut-brain interactions. Mech Aging Dev (2014) 13(6–137):59–69. 10.1016/j.mad.2013.11.011 24333791

[151] Daulatzai MA. Chronic functional bowel syndrome enhances gut-brain axis dysfunction, neuroinflammation, cognitive impairment, and vulnerability to dementia. Neurochem Res (2014) 39:624–44. 10.1007/s11064-014-1266-6 24590859

[152] Burokas A Moloney RD Dinan TG Cryan JF. Microbiota regulation of the mammalian gut-brain axis. Adv Appl Microbiol (2015) 91:1–62. 10.1016/bs.aambs.2015.02.001 25911232

[153] Bhattacharjee S Lukiw WJ Alzheimer’s disease and the microbiome. Front Cell Neurosci (2013) 7:153 10.3389/fncel.2013.00153 24062644 PMC3775450

[154] Hufnagel DA Tukel C Chapman MR Disease to dirt: the biology of microbial amyloids. PLoS Pathog (2013) 9:e1003740 10.1371/journal.ppat.1003740 24278013 PMC3836715

[155] Friedland RP. Mechanisms of molecular mimicry involving the microbiota in neurodegeneration. J Alzheimers Dis (2015) 45:349–62. 10.3233/JAD-142841 25589730

[156] Shoemark DK Allen SJ. The microbiome and disease: reviewing the links between the oral microbiome, aging, and Alzheimer’s disease. J Alzheimers Dis (2015) 43(3):725–38. 10.3233/JAD-141170 25125469

[157] Miklossy J Alzheimer’s disease – a neurospirochetosis. Analysis of the evidence following Koch’s and Hill’s criteria. J Neuroinflammation (2011) 8:90 10.1186/1742-2094-8-90 21816039 PMC3171359

[158] Houeland G Romani A Marchetti C Amato G Capsoni S Cattaneo A Transgenic mice with chronic NGF deprivation and Alzheimer’s disease-like pathology display hippocampal region-specific impairments in short- and long-term plasticities. J Neurosci (2010) 30:13089–94. 10.1523/JNEUROSCI.0457-10.2010 20881126 PMC6633528

[159] Calabrese V Santoro A Monti D Crupi R Di Paola R Latteri S Aging and Parkinson’s disease: inflammaging, neuroinflammation and biological remodeling as key factors in pathogenesis. Free Radic Biol Med (2018) 115:80–91. 10.1016/j.freeradbiomed.2017.10.379 29080843

[160] Buchman AS Shulman JM Nag S Leurgans SE Arnold SE Morris MC Nigral pathology and parkinsonian signs in elders without Parkinson disease. Ann Neurol (2012) 71(2):258–66. 10.1002/ana.22588 22367997 PMC3367476

[161] Olanow CW Do prions cause Parkinson disease? The evidence accumulates. Ann Neurol (2014) 75(3):331–3. 10.1002/ana.24098 24615832

[162] Lee HJ Bae EJ Lee SJ Extracellular α-synuclein – a novel and crucial factor in Lewy body diseases. Nat Rev Neurol (2014) 10(2):92–8. 10.1038/nrneurol.2013.275 24468877

[163] Guo JL Lee VM. Cell-to-cell transmission of pathogenic proteins in neurodegenerative diseases. Nat Med (2014) 20(2):130–8. 10.1038/nm.3457 24504409 PMC4011661

[164] Codolo G Plotegher N Pozzobon T Brucale M Tessari I Bubacco L Triggering of inflammasome by aggregated α-synuclein, an inflammatory response in synucleinopathies. PLoS One (2013) 8(1):e55375. 10.1371/journal.pone.0055375 23383169 PMC3561263

[165] Domert J Rao SB Agholme L Brorsson AC Marcusson J Hallbeck M Spreading of amyloid-β peptides via neuritic cell-to-cell transfer is dependent on insufficient cellular clearance. Neurobiol Dis (2014) 65:82–92. 10.1016/j.nbd.2013.12.019 24412310

[166] Evatt ML Parkinson disease: low vitamin D and Parkinson disease – a causal conundrum. Nat Rev Neurol (2014) 10:8–9. 10.1038/nrneurol.2013.252 24296656

[167] Tchkonia T Zhu Y van Deursen J Campisi J Kirkland JL. Cellular senescence and the senescent secretory phenotype: therapeutic opportunities. J Clin Invest (2013) 123(3):966–72. 10.1172/JCI64098 23454759 PMC3582125

[168] O’Mahony SM Clarke G Borre YE Dinan TG Cryan JF. Serotonin, tryptophan metabolism and the brain-gut-microbiome axis. Behav Brain Res (2015) 277:32–48. 10.1016/j.bbr.2014.07.027 25078296

[169] Clarke G Stilling RM Kennedy PJ Stanton C Cryan JF Dinan TG. Minireview: gut microbiota: the neglected endocrine organ. Mol Endocrinol (2014) 28(8):1221–38. 10.1210/me.2014-1108 24892638 PMC5414803

[170] Mulak A Bonaz B Brain-gut-microbiota axis in Parkinson’s disease. World J Gastroenterol (2015) 21(37):10609–20. 10.3748/wjg.v21.i37.10609 26457021 PMC4588083

[171] Unger MM Spiegel J Dillmann KU Grundmann D Philippeit H Bürmann J Short chain fatty acids and gut microbiota differ between patients with Parkinson’s disease and age-matched controls. Parkinsonism Relat Disord (2016) 32:66–72. 10.1016/j.parkreldis.2016.08.019 27591074

[172] Sampson TR Debelius JW Thron T Janssen S Shastri GG Ilhan ZE Gut microbiota regulate motor deficits and neuroinflammation in a model of Parkinson’s disease. Cell (2016) 167(6):1469.e–80.e. 10.1016/j.cell.2016.11.018 27912057 PMC5718049

[173] Santoro A Ostan R Candela M Biagi E Brigidi P Capri M Gut microbiota changes in the extreme decades of human life: a focus on centenarians. Cell Mol Life Sci (2017) 75(1):129–48. 10.1007/s00018-017-2674-y 29032502 PMC5752746

[174] Biagi E Nylund L Candela M Ostan R Bucci L Pini E Through aging, and beyond: gut microbiota and inflammatory status in seniors and centenarians. PLoS One (2010) 5(5):e10667 10.1371/journal.pone.0010667 Erratum in: PLoS One (2010) 5(6). 10.1371/journal.pone.0010667 20498852 PMC2871786

[175] Gao J Xu K Liu H Liu G Bai M Peng C Impact of the gut microbiota on intestinal immunity mediated by tryptophan metabolism. Front Cell Infect Microbiol (2018) 8:13. 10.3389/fcimb.2018.00013 29468141 PMC5808205

[176] Rampelli S Candela M Turroni S Biagi E Collino S Franceschi C Functional metagenomic profiling of intestinal microbiome in extreme aging. Aging (Albany NY) (2013) 5(12):902–12. 10.18632/aging.100623 24334635 PMC3883706

[177] Franceschi C Campisi J. Chronic inflammation (inflammaging) and its potential contribution to age-associated diseases. J Gerontol A Biol Sci Med Sci (2014) 69(Suppl 1):S4–9. 10.1093/gerona/glu057 24833586

[178] Stone TW Darlington LG. The kynurenine pathway as a therapeutic target in cognitive and neurodegenerative disorders. Br J Pharmacol (2013) 169(6):1211–27. 10.1111/bph.12230 23647169 PMC3831703

[179] Maddison DC Giorgini F. The kynurenine pathway and neurodegenerative disease. Semin Cell Dev Biol (2015) 40:134–41. 10.1016/j.semcdb.2015.03.002 25773161

[180] Pereira PAB Aho VTE Paulin L Pekkonen E Auvinen P Scheperjans F. Oral and nasal microbiota in Parkinson’s disease. Parkinsonism Relat Disord (2017) 38:61–7. 10.1016/j.parkreldis.2017.02.026 28259623

[181] Feldman M Brennan FM Maini RN Role of cytokines in rheumatoid arthritis. Annu Rev Immunol (1996) 43:28–38. 10.1146/annurev.immunol.14.1.397 8717520

[182] Taniguchi N Kawahara K Yone K Hashiguchi T Yamakuchi M Goto M High mobility group box chromosomal protein 1 plays a role in the pathogenesis of rheumatoid arthritis as a novel cytokine. Arthritis Rheum (2003) 48(4):971–81. 10.1002/art.10859 12687539

[183] Calder PC Bosco N Bourdet-Sicard R Capuron L Delzenne N Doré J Health relevance of the modification of low grade inflammation in aging (inflammaging) and the role of nutrition. Aging Res Rev (2017) 40:95–119. 10.1016/j.arr.2017.09.001 28899766

[184] Taniguchi N Caramés B Ronfani L Ulmer U Komiya S Bianchi ME Aging-related loss of the chromatin protein HMGB2 in articular cartilage is linked to reduced cellularity and osteoarthritis. Proc Natl Acad Sci U S A (2009) 106(4):1181–6. 10.1073/pnas.0806062106 19139395 PMC2633567

[185] Amin AR Islam AB. Genomic analysis and differential expression of HMG and S100A family in human arthritis: upregulated expression of chemokines, IL-8 and nitric oxide by HMGB1. DNA Cell Biol (2014) 33:550–65. 10.1089/dna.2013.2198 24905701 PMC4117271

[186] Ali T Lam D Bronze MS Humphrey MB. Osteoporosis in inflammatory bowel disease. Am J Med (2009) 122:599–604. 10.1016/j.amjmed.2009.01.022 19559158 PMC2894700

[187] Wohl Y Dreiher J Cohen AD. Pemphigus and osteoporosis: a case-control study. Arch Dermatol (2010) 146(10):1126–31. 10.1001/archdermatol.2010.257 20956645

[188] Kampman MT Eriksen EF Holmoy T. Multiple sclerosis, a cause of secondary osteoporosis? What is the evidence and what are the clinical implications? Acta Neurol Scand Suppl (2011) 124(191):44–9. 10.1111/j.1600-0404.2011.01543.x 21711256

[189] Bultink IE Osteoporosis and fractures in systemic lupus erythematosus. Arthritis Care Res (Hoboken) (2012) 64:2–8. 10.1002/acr.20568 22213721

[190] Bultink IE Vis M van der Horst-Bruinsma IE Lems WF Inflammatory rheumatic disorders and bone. Curr Rheumatol Rep (2012) 14:224–30. 10.1007/s11926-012-0252-8 22477520 PMC3338911

[191] Sambrook PN Geusens P. The epidemiology of osteoporosis and fractures in ankylosing spondylitis. Ther Adv Musculoskelet Dis (2012) 4:287–92. 10.1177/1759720X12441276 22859927 PMC3403252

[192] Keller JJ Kang JH Lin HC. Association between osteoporosis and psoriasis: results from the longitudinal health insurance database in Taiwan. Osteoporos Int (2013) 24:1835–41. 10.1007/s00198-012-2185-5 23052942

[193] Faienza MF Ventura A Marzano F Cavallo L. Postmenopausal osteoporosis: the role of immune system cells. Clin Dev Immunol (2013) 2013:575936. 10.1155/2013/575936 23762093 PMC3677008

[194] Schett G Kiechl S Weger S Pederiva A Mayr A Petrangeli M High-sensitivity C-reactive protein and risk of nontraumatic fractures in the Bruneck study. Arch Intern Med (2006) 166:2495–501. 10.1001/archinte.166.22.2495 17159016

[195] Eriksson AL Moverare-Skrtic S Ljunggren O Karlsson M Mellstrom D Ohlsson C. High-sensitivity CRP is an independent risk factor for all fractures and vertebral fractures in elderly men: the MrOS Sweden study. J Bone Miner Res (2014) 29:418–23. 10.1002/jbmr.2037 23857741 PMC4238816

[196] Ding C Parameswaran V Udayan R Burgess J Jones G. Circulating levels of inflammatory markers predict change in bone mineral density and resorption in older adults: a longitudinal study. J Clin Endocrinol Metab (2008) 93:1952–8. 10.1210/jc.2007-2325 18285417

[197] Gehrs KM Jackson JR Brown EN Allikmets R Hageman GS. Complement, age-related macular degeneration and a vision of the future. Arch Ophthalmol (2010) 128(3):349–58. 10.1001/archophthalmol.2010.18 20212207 PMC4405117

[198] Gallenga CE Parmeggiani F Costagliola C Sebastiani A Gallenga PE. Inflammaging: should this term be suitable for age related macular degeneration too? Inflamm Res (2014) 63(2):105–7. 10.1007/s00011-013-0684-2 24202618

[199] Zhuang Y Lyga J. Inflammaging in skin and other tissues – the roles of complement system and macrophage. Inflamm Allergy Drug Targets (2014) 13(3):153–61. 10.2174/1871528113666140522112003 24853681 PMC4082166

[200] Chen M Xu H. Parainflammation, chronic inflammation, and age-related macular degeneration. J Leukoc Biol (2015) 98(5):713–25. 10.1189/jlb.3RI0615-239R 26292978 PMC4733662

[201] Ferrington DA Sinha D Kaarniranta K. Defects in retinal pigment epithelial cell proteolysis and the pathology associated with age-related macular degeneration. Prog Retin Eye Res (2016) 51:69–89. 10.1016/j.preteyeres.2015.09.002 26344735 PMC4769684

[202] Kaarniranta K Salminen A Eskelinen EL Kopitz J. Heat shock proteins as gatekeepers of proteolytic pathways—implications for age-related macular degeneration (AMD). Aging Res Rev (2009) 8(2):128–39. 10.1016/j.arr.2009.01.001 19274853

[203] Ambati J Atkinson JP Gelfand BD. Immunology of age-related macular degeneration. Nat Rev Immunol (2013) 13(6):438–51. 10.1038/nri3459 23702979 PMC3941009

[204] Maraldi NM Capanni C Cenni V Fini M Lattanzi G. Laminopathies and lamin-associated signaling pathways. J Cell Biochem (2011) 112(4):979–92. 10.1002/jcb.22992 21400569

[205] Baird PA Sadovnick AD. Life expectancy in Down syndrome adults. Lancet (1988) 2(8624):1354–6. 10.1016/S0140-6736(88)90881-1 2904063

[206] Janicki MP Dalton AJ Henderson CM Davidson PW. Mortality and morbidity among older adults with intellectual disability: health services considerations. Disabil Rehabil (1999) 21(5–6):284–94. 10.1080/096382899297710 10381241

[207] Bittles AH Glasson EJ Clinical, social, and ethical implications of changing life expectancy in Down syndrome. Dev Med Child Neurol (2004) 46(4):282–6. 10.1017/S0012162204000441 15077706

[208] Glasson EJ Sullivan SG Hussain R Petterson BA Montgomery PD Bittles AH The changing survival profile of people with Down’s syndrome: implications for genetic counseling. Clin Genet (2002) 62(5):390–3. 10.1034/j.1399-0004.2002.620506.x 12431254

[209] Coppus AM Evenhuis HM Verberne GJ Visser FE Oostra BA Eikelenboom P Survival in elderly persons with Down syndrome. J Am Geriatr Soc (2008) 56(12):2311–6. 10.1111/j.1532-5415.2008.01999.x 19093931

[210] Lott IT. Neurological phenotypes for Down syndrome across the life span. Prog Brain Res (2012) 197:101–21. 10.1016/B978-0-444-54299-1.00006-6 22541290 PMC3417824

[211] Arvio M Luostarinen L. Down syndrome in adults: a 27-year follow-up of adaptive skills. Clin Genet (2016) 90(5):456–60. 10.1111/cge.12787 27067497

[212] Colacurcio DJ Pensalfini A Jiang Y Nixon RA Dysfunction of autophagy and endosomal-lysosomal pathways: roles in pathogenesis of Down syndrome and Alzheimer’s disease. Free Radic Biol Med (2017) 114:40–51. 10.1016/j.freeradbiomed.2017.10.001 28988799 PMC5748263

[213] Lauritzen I Pardossi-Piquard R Bourgeois A Pagnotta S Biferi MG Barkats M Intraneuronal aggregation of the β-CTF fragment of APP (C99) induces Aβ-independent lysosomal-autophagic pathology. Acta Neuropathol (2016) 132(2):257–76. 10.1007/s00401-016-1577-6 27138984 PMC4947121

[214] Cossarizza A Ortolani C Forti E Montagnani G Paganelli R Zannotti M Age-related expansion of functionally inefficient cells with markers of natural killer activity in Down’s syndrome. Blood (1991) 77(6):1263–70. 1825795

[215] Jenkins EC Velinov MT Ye L Gu H Li S Jenkins EC Jr Telomere shortening in T lymphocytes of older individuals with Down syndrome and dementia. Neurobiol Aging (2006) 27(7):941–5. 10.1016/j.neurobiolaging.2005.05.021 16046031

[216] Park E Alberti J Mehta P Dalton A Sersen E Schuller-Levis G. Partial impairment of immune functions in peripheral blood leukocytes from aged men with Down’s syndrome. Clin Immunol (2000) 95(1 Pt 1):62–9. 10.1006/clim.2000.4834 10794433

[217] Cuadrado E Barrena MJ. Immune dysfunction in Down’s syndrome: primary immune deficiency or early senescence of the immune system? Clin Immunol Immunopathol (1996) 78(3):209–14. 10.1006/clin.1996.0031 8605695

[218] Kusters MA Verstegen RH de Vries E. Down syndrome: is it really characterized by precocious immunosenescence? Aging Dis (2011) 2(6):538–45. 22396900 PMC3295065

[219] Trotta MB Serro Azul JB Wajngarten M Fonseca SG Goldberg AC Kalil JE. Inflammatory and immunological parameters in adults with Down syndrome. Immun Aging (2011) 8(1):4. 10.1186/1742-4933-8-4 21496308 PMC3101128

[220] Iulita MF Ower A Barone C Pentz R Gubert P Romano C An inflammatory and trophic disconnect biomarker profile revealed in Down syndrome plasma: relation to cognitive decline and longitudinal evaluation. Alzheimers Dement (2016) 12(11):1132–48. 10.1016/j.jalz.2016.05.001 27452424

[221] Arbuzova S Hutchin T Cuckle H Mitochondrial dysfunction and Down’s syndrome. Bioessays (2002) 24:681–4. 10.1002/bies.10138 12210526

[222] Izzo A Nitti M Mollo N Paladino S Procaccini C Faicchia D Metformin restores the mitochondrial network and reverses mitochondrial dysfunction in Down syndrome cells. Hum Mol Genet (2017) 26(6):1056–69. 10.1093/hmg/ddx016 28087733

[223] Horvath S Garagnani P Bacalini MG Pirazzini C Salvioli S Gentilini D Accelerated epigenetic aging in Down syndrome. Aging Cell (2015) 14(3):491–5. 10.1111/acel.12325 25678027 PMC4406678

[224] Borelli V Vanhooren V Lonardi E Reiding KR Capri M Libert C Plasma N-glycome signature of Down syndrome. J Proteome Res (2015) 14(10):4232–45. 10.1021/acs.jproteome.5b00356 26334954

[225] Franceschi C Garagnani P Suggestions from geroscience for the genetics of age-related diseases. PLoS Genet (2016) 12(11):e1006399 10.1371/journal.pgen.1006399 27832069 PMC5104376

[226] Fraga MF Ballestar E Paz MF Ropero S Setien F Ballestar ML Epigenetic differences arise during the lifetime of monozygotic twins. Proc Natl Acad Sci U S A (2005) 102(30):10604–9. 10.1073/pnas.0500398102 16009939 PMC1174919

[227] Bacalini MG Gentilini D Boattini A Giampieri E Pirazzini C Giuliani C Identification of a DNA methylation signature in blood cells from persons with Down Syndrome. Aging (Albany NY) (2015) 7(2):82–96. 10.18632/aging.100715 25701644 PMC4359691

[228] Durso DF Bacalini MG do Valle ÍF Pirazzini C Bonafé M Castellani G Aberrant methylation patterns in colorectal cancer: a meta-analysis. Oncotarget (2017) 8(8):12820–30. 10.18632/oncotarget.14590 28086223 PMC5355058

[229] Garagnani P Bacalini MG Pirazzini C Gori D Giuliani C Mari D Methylation of ELOVL2 gene as a new epigenetic marker of age. Aging Cell (2012) 11(6):1132–4. 10.1111/acel.12005 23061750

[230] Horvath S DNA methylation age of human tissues and cell types. Genome Biol. 2013;14(10):R115. Genome Biol (2015) 16:96 10.1186/gb-2013-14-10-r115 24138928 PMC4015143

[231] Hannum G Guinney J Zhao L Zhang L Hughes G Sadda S Genome-wide methylation profiles reveal quantitative views of human aging rates. Mol Cell (2013) 49(2):359–67. 10.1016/j.molcel.2012.10.016 23177740 PMC3780611

[232] Gentilini D Garagnani P Pisoni S Bacalini MG Calzari L Mari D Stochastic epigenetic mutations (DNA methylation) increase exponentially in human aging and correlate with X chromosome inactivation skewing in females. Aging (Albany NY) (2015) 7(8):568–78. 10.18632/aging.100792 26342808 PMC4586102

[233] Shi L Jiang F Ouyang F Zhang J Wang Z Shen X DNA methylation markers in combination with skeletal and dental ages to improve age estimation in children. Forensic Sci Int Genet (2017) 33:1–9. 10.1016/j.fsigen.2017.11.005 29172065

[234] Jung SE Shin KJ Lee HY. DNA methylation-based age prediction from various tissues and body fluids. BMB Rep (2017) 50(11):546–53. 10.5483/BMBRep.2017.50.11.175 28946940 PMC5720467

[235] Giuliani C Cilli E Bacalini MG Pirazzini C Sazzini M Gruppioni G Inferring chronological age from DNA methylation patterns of human teeth. Am J Phys Anthropol (2016) 159(4):585–95. 10.1002/ajpa.22921 26667772

[236] Weidner CI Lin Q Koch CM Eisele L Beier F Ziegler P Aging of blood can be tracked by DNA methylation changes at just three CpG sites. Genome Biol (2014) 15(2):R24. 10.1186/gb-2014-15-2-r24 24490752 PMC4053864

[237] Levine ME Hosgood HD Chen B Absher D Assimes T Horvath S DNA methylation age of blood predicts future onset of lung cancer in the women’s health initiative. Aging (Albany NY) (2015) 7(9):690–700. 10.18632/aging.100809 26411804 PMC4600626

[238] Durso DF Bacalini MG Sala C Pirazzini C Marasco E Bonafé M Acceleration of leukocytes’ epigenetic age as an early tumor and sex-specific marker of breast and colorectal cancer. Oncotarget (2017) 8(14):23237–45. 10.18632/oncotarget.15573 28423572 PMC5410300

[239] Horvath S Ritz BR. Increased epigenetic age and granulocyte counts in the blood of Parkinson’s disease patients. Aging (Albany NY) (2015) 7(12):1130–42. 10.18632/aging.100859 26655927 PMC4712337

[240] Levine ME Lu AT Bennett DA Horvath S Epigenetic age of the pre-frontal cortex is associated with neuritic plaques, amyloid load, and Alzheimer’s disease related cognitive functioning. Aging (Albany NY) (2015) 7(12):1198–211. 10.18632/aging.100864 26684672 PMC4712342

[241] Levine AJ Quach A Moore DJ Achim CL Soontornniyomkij V Masliah E Accelerated epigenetic aging in brain is associated with pre-mortem HIV-associated neurocognitive disorders. J Neurovirol (2016) 22(3):366–75. 10.1007/s13365-015-0406-3 26689571 PMC4900944

[242] Maierhofer A Flunkert J Oshima J Martin GM Haaf T Horvath S. Accelerated epigenetic aging in Werner syndrome. Aging (Albany NY) (2017) 9(4):1143–52. 10.18632/aging.101217 28377537 PMC5425119

[243] Marioni RE Shah S McRae AF Chen BH Colicino E Harris SE DNA methylation age of blood predicts all-cause mortality in later life. Genome Biol (2015) 16:25. 10.1186/s13059-015-0584-6 25633388 PMC4350614

[244] Perna L Zhang Y Mons U Holleczek B Saum KU Brenner H. Epigenetic age acceleration predicts cancer, cardiovascular, and all-cause mortality in a German case cohort. Clin Epigenetics (2016) 8:64. 10.1186/s13148-016-0228-z 27274774 PMC4891876

[245] Horvath S Pirazzini C Bacalini MG Gentilini D Di Blasio AM Delledonne M Decreased epigenetic age of PBMCs from Italian semi supercentenarians and their offspring. Aging (Albany NY) (2015) 7(12):1159–70. 10.18632/aging.100861 26678252 PMC4712339

[246] Armstrong NJ Mather KA Thalamuthu A Wright MJ Trollor JN Ames D Aging, exceptional longevity and comparisons of the Hannum and Horvath epigenetic clocks. Epigenomics (2017) 9(5):689–700. 10.2217/epi-2016-0179 28470125

[247] Miyahara K Nouso K Dohi C Morimoto Y Kinugasa H Wada N Alteration of N-glycan profiles in patients with chronic hepatitis and hepatocellular carcinoma. Hepatol Res (2014) 45:986–93. 10.1111/hepr.12441 25495282

[248] Blomme B Fitzpatrick E Quaglia A De Bruyne R Dhawan A Van Vlierberghe H Serum protein N-glycosylation in paediatric non-alcoholic fatty liver disease. Pediatr Obes (2012) 7(2):165–73. 10.1111/j.2047-6310.2011.00024.x 22434757

[249] Blomme B Francque S Trépo E Libbrecht L Vanderschaeghe D Verrijken A N-glycan based biomarker distinguishing non-alcoholic steatohepatitis from steatosis independently of fibrosis. Dig Liver Dis (2012) 44(4):315–22. 10.1016/j.dld.2011.10.015 22119618

[250] Keser T Gornik I Vučković F Selak N Pavić T Lukić E Increased plasma N-glycome complexity is associated with higher risk of type 2 diabetes. Diabetologia (2017) 60(12):2352–60. 10.1007/s00125-017-44269 28905229

[251] Lemmers RFH Vilaj M Urda D Agakov F Šimurina M Klaric L IgG glycan patterns are associated with type 2 diabetes in independent European populations. Biochim Biophys Acta (2017) 1861(9):2240–9. 10.1016/j.bbagen.2017.06.020 28668296

[252] Inafuku S Noda K Amano M Ohashi T Yoshizawa C Saito W Alteration of N-glycan profiles in diabetic retinopathy. Invest Ophthalmol Vis Sci (2015) 56(9):5316–22. 10.1167/iovs.15-16747 26258617

[253] Testa R Vanhooren V Bonfigli AR Boemi M Olivieri F Ceriello A N-glycomic changes in serum proteins in type 2 diabetes mellitus correlate with complications and with metabolic syndrome parameters. PLoS One (2015) 10(3):e0119983. 10.1371/journal.pone.0119983 25793407 PMC4368037

[254] de Kreutzenberg SV Ceolotto G Cattelan A Pagnin E Mazzucato M Garagnani P Metformin improves putative longevity effectors in peripheral mononuclear cells from subjects with prediabetes. A randomized controlled trial. Nutr Metab Cardiovasc Dis (2015) 25(7):686–93. 10.1016/j.numecd.2015.03.007 25921843

[255] Huang C Liu Y Wu H Sun D Li Y Characterization of IgG glycosylation in rheumatoid arthritis patients by MALDI-TOF-MS(n) and capillary electrophoresis. Anal Bioanal Chem (2017) 409(15):3731–9. 10.1007/s00216-017-0302-1 28397166

[256] Gińdzieńska-Sieśkiewicz E Radziejewska I Domysławska I Klimiuk PA Sulik A Rojewska J Changes of glycosylation of IgG in rheumatoid arthritis patients treated with methotrexate. Adv Med Sci (2016) 61(2):193–7. 10.1016/j.advms.2015.12.009 26876088

[257] Nakagawa H Hato M Takegawa Y Deguchi K Ito H Takahata M Detection of altered N-glycan profiles in whole serum from rheumatoid arthritis patients. J Chromatogr B Analyt Technol Biomed Life Sci (2007) 853(1–2):133–7. 10.1016/j.jchromb.2007.03.003 17392038

[258] Field MC Amatayakul-Chantler S Rademacher TW Rudd PM Dwek RA. Structural analysis of the N-glycans from human immunoglobulin A1: comparison of normal human serum immunoglobulin A1 with that isolated from patients with rheumatoid arthritis. Biochem J (1994) 299(Pt 1):261–75. 10.1042/bj2990261 8166649 PMC1138050

[259] Tanaka T Yoneyama T Noro D Imanishi K Kojima Y Hatakeyama S Aberrant N-glycosylation profile of serum immunoglobulins is a diagnostic biomarker of urothelial carcinomas. Int J Mol Sci (2017) 18(12):E2632. 10.3390/ijms18122632 29210993 PMC5751235

[260] Qin R Zhao J Qin W Zhang Z Zhao R Han J Discovery of non-invasive glycan biomarkers for detection and surveillance of gastric cancer. J Cancer (2017) 8(10):1908–16. 10.7150/jca.17900 28819389 PMC5556655

[261] Wang M Fang M Zhu J Feng H Warner E Yi C Serum N-glycans outperform CA19-9 in diagnosis of extrahepatic cholangiocarcinoma. Electrophoresis (2017) 38(21):2749–56. 10.1002/elps.201700084 28752594

[262] Liu T Shang S Li W Qin X Sun L Zhang S Assessment of hepatocellular carcinoma metastasis glycobiomarkers using advanced quantitative N-glycoproteome analysis. Front Physiol (2017) 8:472. 10.3389/fphys.2017.00472 28736531 PMC5500640

[263] Vanhooren V Dewaele S Kuro-O M Taniguchi N Dollé L van Grunsven LA Alteration in N-glycomics during mouse aging: a role for FUT8. Aging Cell (2011) 10(6):1056–66. 10.1111/j.1474-9726.2011.00749.x 21951615

[264] Ruhaak LR Uh HW Beekman M Hokke CH Westendorp RG Houwing-Duistermaat J Plasma protein N-glycan profiles are associated with calendar age, familial longevity and health. J Proteome Res (2011) 10(4):1667–74. 10.1021/pr1009959 21184610

[265] Ruhaak LR Koeleman CA Uh HW Stam JC van Heemst D Maier AB Targeted biomarker discovery by high throughput glycosylation profiling of human plasma alpha1-antitrypsin and immunoglobulin A. PLoS One (2013) 8(9):e73082. 10.1371/journal.pone.0073082 24039863 PMC3767703

[266] Dall’Olio F Vanhooren V Chen CC Slagboom PE Wuhrer M Franceschi C. N-glycomic biomarkers of biological aging and longevity: a link with inflammaging. Aging Res Rev (2013) 12(2):685–98. 10.1016/j.arr.2012.02.002 22353383

[267] Biagi E Franceschi C Rampelli S Severgnini M Ostan R Turroni S Gut microbiota and extreme longevity. Curr Biol (2016) 26(11):1480–5. 10.1016/j.cub.2016.04.016 27185560

[268] Thevaranjan N Puchta A Schulz C Naidoo A Szamosi JC Verschoor CP Age-associated microbial dysbiosis promotes intestinal permeability, systemic inflammation, and macrophage dysfunction. Cell Host Microbe (2017) 21(4):455.e–66.e. 10.1016/j.chom.2017.03.002 28407483 PMC5392495

[269] Maffei VJ Kim S Blanchard E IV Luo M Jazwinski SM Taylor CM Biological aging and the human gut microbiota. J Gerontol A Biol Sci Med Sci (2017) 72(11):1474–82. 10.1093/gerona/glx042 28444190 PMC5861892

[270] Montoliu I Scherer M Beguelin F DaSilva L Mari D Salvioli S Serum profiling of healthy aging identifies phospho- and sphingolipid species as markers of human longevity. Aging (Albany NY) (2014) 6(1):9–25. 10.18632/aging.100630 24457528 PMC3927806

[271] Olivieri F Capri M Bonafè M Morsiani C Jung HJ Spazzafumo L Circulating miRNAs and miRNA shuttles as biomarkers: perspective trajectories of healthy and unhealthy aging. Mech Aging Dev (2017) 165(Pt B):162–70. 10.1016/j.mad.2016.12.004 27986629 PMC5481482

[272] Pinti M Cevenini E Nasi M De Biasi S Salvioli S Monti D Circulating mitochondrial DNA increases with age and is a familiar trait: implications for “inflamm-aging”. Eur J Immunol (2014) 44(5):1552–62. 10.1002/eji.201343921 24470107

[273] Gems D. The aging-disease false dichotomy: understanding senescence as pathology. Front Genet (2015) 6:212. 10.3389/fgene.2015.00212 26136770 PMC4468941

[274] Rattan SI. Aging is not a disease: implications for intervention. Aging Dis (2014) 5(3):196–202. 10.14336/AD.2014.0500196 24900942 PMC4037311

[275] Gladyshev TV Gladyshev VN. A disease or not a disease? Aging as a pathology. Trends Mol Med (2016) 22(12):995–6. 10.1016/j.molmed.2016.09.009 27793599 PMC5540438

[276] Baar MP Van Willigenburg H de Keizer PLJ Maintenance and repair of an aging life cycle. Oncotarget (2017) 8(50):86985–6. 10.18632/oncotarget.18046 29152057 PMC5675609

[277] Longo VD Antebi A Bartke A Barzilai N Brown-Borg HM Caruso C Interventions to slow aging in humans: are we ready? Aging Cell (2015) 14(4):497–510. 10.1111/acel.12338 25902704 PMC4531065

[278] Farr JN Xu M Weivoda MM Monroe DG Fraser DG Onken JL Targeting cellular senescence prevents age-related bone loss in mice. Nat Med (2017) 23(9):1072–9. 10.1038/nm.4385 Erratum in Nat Med (2017) 23(11)1384. 10.1038/nm.4385 28825716 PMC5657592

[279] Ostan R Monti D Gueresi P Bussolotto M Franceschi C Baggio G. Gender, aging and longevity in humans: an update of an intriguing/neglected scenario paving the way to a gender-specific medicine. Clin Sci (Lond) (2016) 130(19):1711–25. 10.1042/CS20160004 27555614 PMC4994139

[280] Franceschi C Motta L Valensin S Rapisarda R Franzone A Berardelli M Do men and women follow different trajectories to reach extreme longevity? Italian multicenter study on centenarians (IMUSCE). Aging (Milano) (2000) 12(2):77–84. 10902049 10.1007/BF03339894

[281] Flak MB Neves JF Blumberg RS Immunology. Welcome to the microgenderome. Science (2013) 339(6123):1044–5. 10.1126/science.1236226 23449586 PMC4005781

[282] Mattson MP. Awareness of hormesis will enhance future research in basic and applied neuroscience. Crit Rev Toxicol (2008) 38(7):633–9. 10.1080/10408440802026406 18709572 PMC2612999

[283] Martucci M Ostan R Biondi F Bellavista E Fabbri C Bertarelli C Mediterranean diet and inflammaging within the hormesis paradigm. Nutr Rev (2017) 75(6):442–55. 10.1093/nutrit/nux013 28595318 PMC5914347

[284] Calabrese EJ. The emergence of the dose-response concept in biology and medicine. Int J Mol Sci (2016) 17(12):E2034. 10.3390/ijms17122034 27929392 PMC5187834

[285] Calabrese EJ Mattson MP. How does hormesis impact biology, toxicology, and medicine? NPJ Aging Mech Dis (2017) 3:13. 10.1038/s41514-017-0013-z 28944077 PMC5601424

[286] Rose G Santoro A Salvioli S. Mitochondria and mitochondria-induced signalling molecules as longevity determinants. Mech Aging Dev (2017) 165(Pt B):115–28. 10.1016/j.mad.2016.12.002 27964991

[287] Fulop T Larbi A Dupuis G Le Page A Frost EH Cohen AA Immunosenescence and inflamm-aging as two sides of the same coin: friends or foes? Front Immunol (2018) 8:1960. 10.3389/fimmu.2017.01960 29375577 PMC5767595

[288] Vijg J Le Bourg E. Aging and the inevitable limit to human life span. Gerontology (2017) 63(5):432–4. 10.1159/000477210 28511176

[289] Grignolio A Franceschi C History of Researches into Aging/Senescence. eLS Online Reference. Chichester (WS), UK: John Wiley & Sons Ltd (2012).

[290] Niebyl PH Old age, fever, and the lamp metaphor. J Hist Med Allied Sci (1971) 26(4):351–68. 10.1093/jhmas/XXVI.4.351 4946289

[291] Schäfer D ‘That senescence itself is an illness’: a transitional medical concept of age and aging in the eighteenth century. Med Hist (2002) 46:525–48. 12408094 PMC1044563

[292] Byl S La gerontologie de Galien. Hist Philos Life Sci (1988) 10:73–92. 3045855

[293] Howell TH Avicenna and his regimen of old age. Age and Aging (1987) 16(1):58–9. 10.1093/ageing/16.1.58 3551552

[294] Grmek MD On Aging and Old Age; Basic Problems and Historic Aspects of Gerontology and Geriatrics. Den Haag: W. Junk (1958).

[295] von Kondratowitz HJ The medicalization of old age. In: Pelling MS Michael R, editors. Life, Death, and the Elderly: Historical Perspectives. London: Routledge (1991). p. 134–64.

